# Supplementary material for: Organic NIR-II molecule with long blood half-life for in vivo dynamic vascular imaging
Source: Nat Commun. 2020 Jun 18;11:3102. doi: 10.1038/s41467-020-16924-z (PMC7303218; doi:10.1038/s41467-020-16924-z)
Supplement: Supplementary file 1 — Supplementary Information [file 41467_2020_16924_MOESM1_ESM.pdf]

Supplementary Information

Organic NIR-II molecule with long blood half-life for in vivo dynamic vascular imaging

Li *et al.*

Supplementary Figures

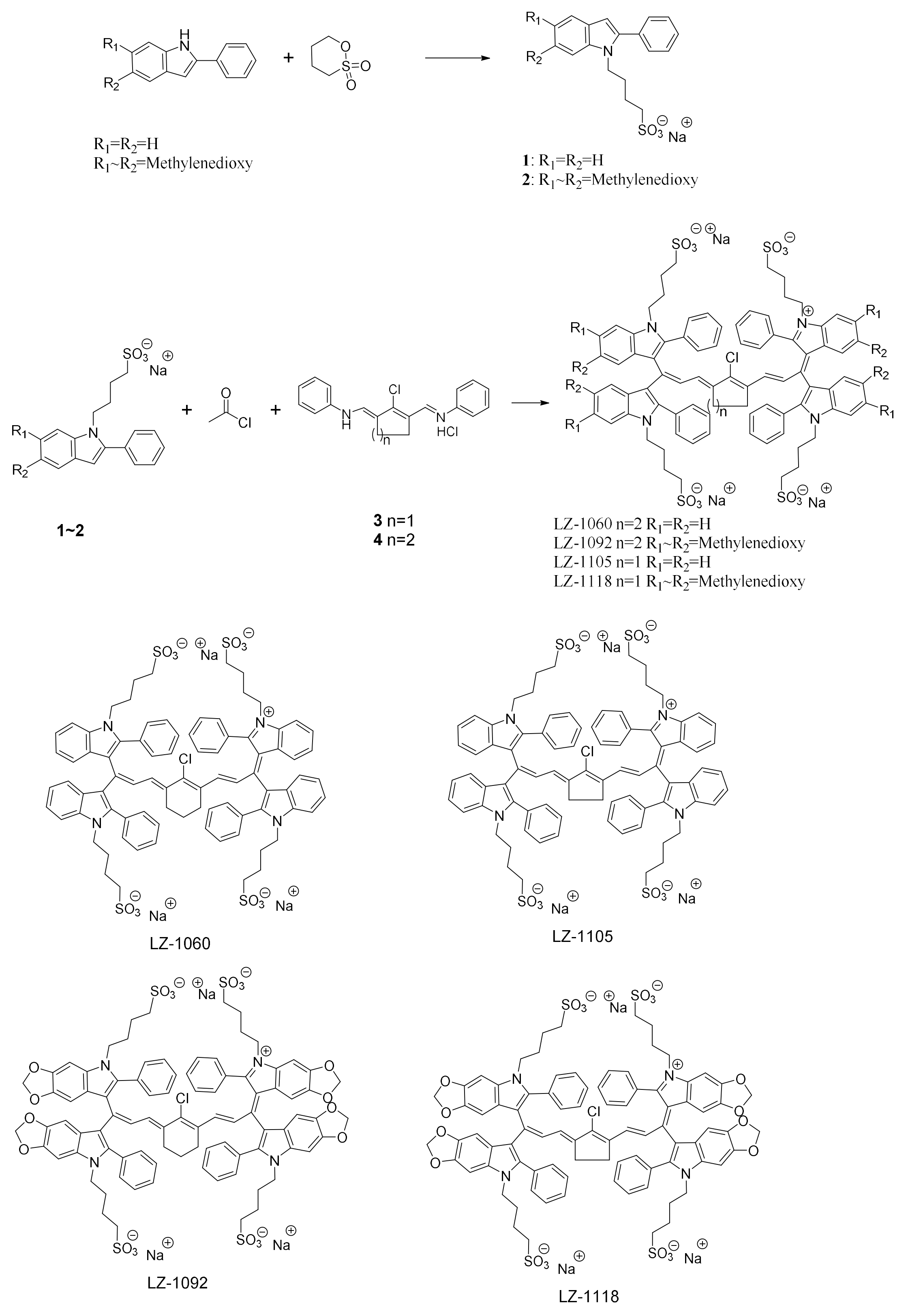

Supplementary Figure 1. Synthesis of compounds LZ dyes.

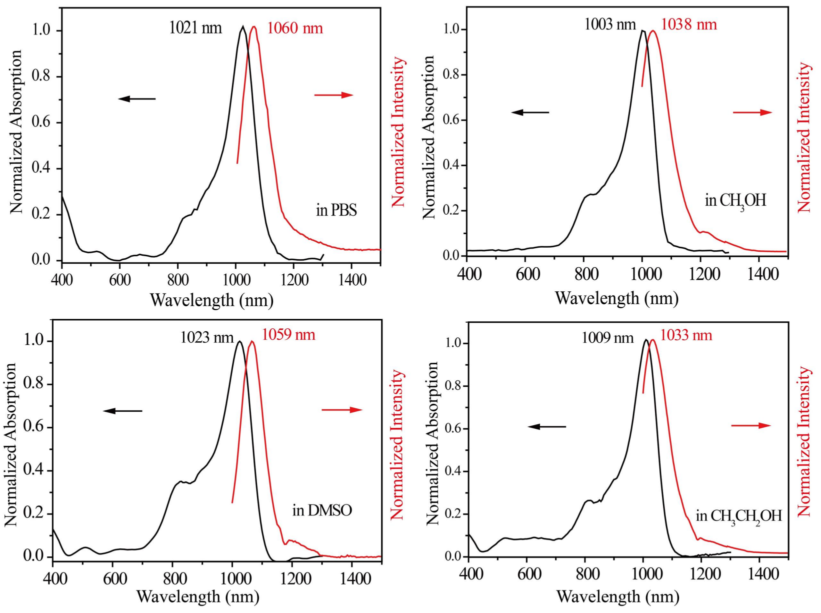

Supplementary Figure 2. Optical characterization of LZ-1060. Absorption and fluorescence emission spectra of LZ-1060 in different solvents as indicated. The fluorescence emission spectra were obtained under 980 nm laser excitation

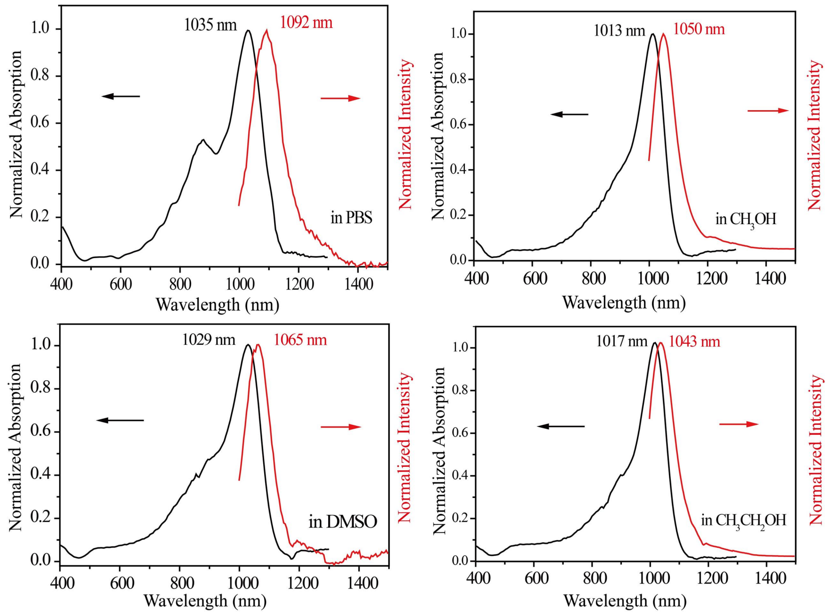

Supplementary Figure 3. Optical characterization of LZ-1092. Absorption and fluorescence emission spectra of LZ-1092 in different solvents as indicated. The fluorescence emission spectra were obtained under 980 nm laser excitation.

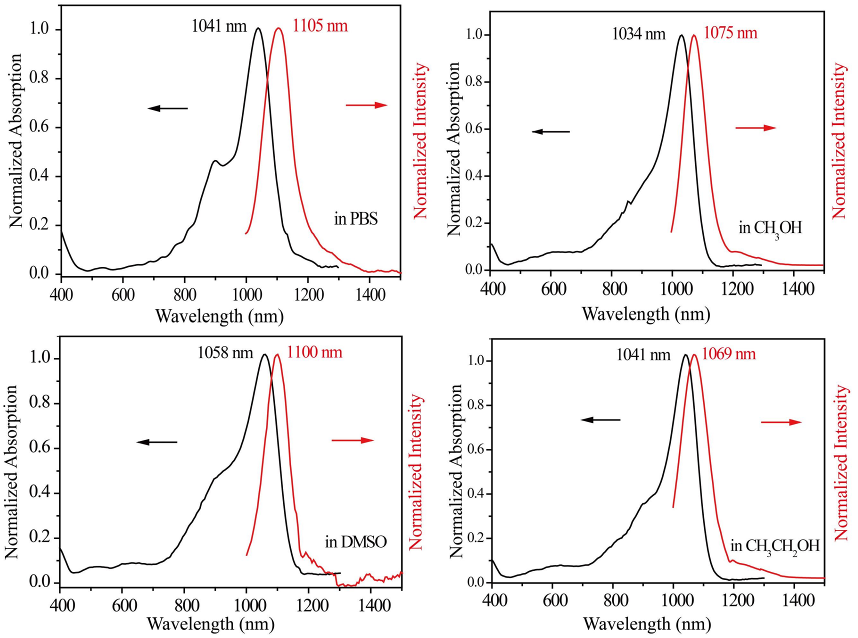

Supplementary Figure 4. Optical characterization of LZ-1105. Absorption and fluorescence emission spectra of LZ-1105 in different solvents as indicated. The fluorescence emission spectra were obtained under 980 nm laser excitation.

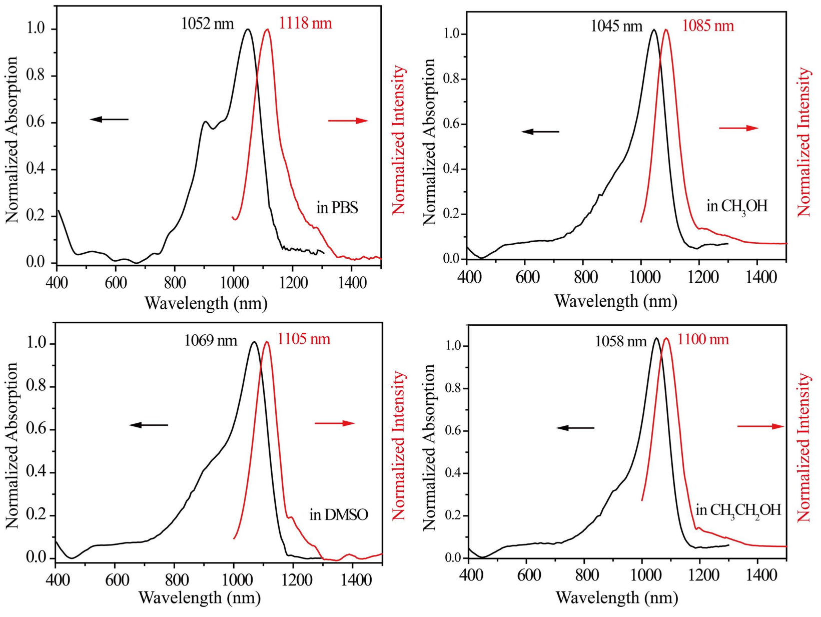

Supplementary Figure 5. Optical characterization of LZ-1118. Absorption and fluorescence emission spectra of LZ-1118 in different solvents as indicated. The fluorescence emission spectra were obtained under 980 nm laser excitation.

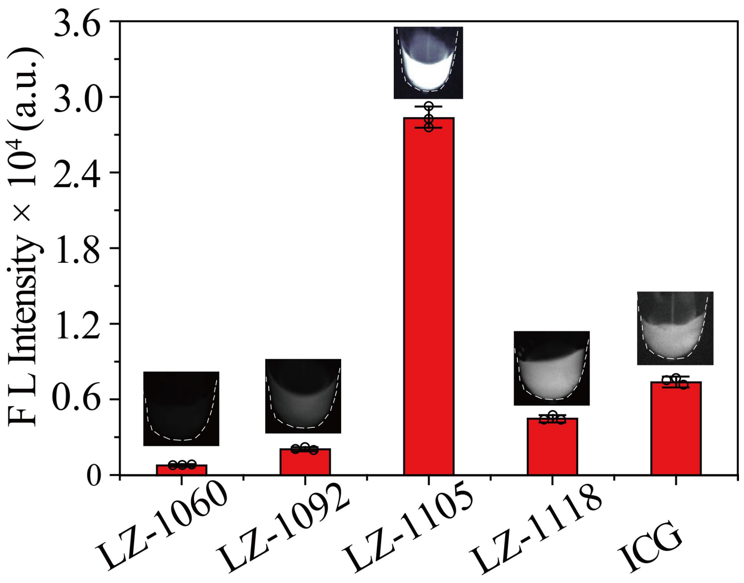

Supplementary Figure 6. Optical characterization of LZ dyes and ICG in mice blood. The fluorescence intensity of LZ series dyes and ICG in mice blood detected by the InGaAs camera (1064 nm excitation for LZ dyes, 30 mW cm^{-2}, 1400 nm long-pass filter; 808 nm excitation for ICG, 30 mW cm^{-2}, 1300 nm long-pass filter). Insets: NIR-II fluorescence images of LZ dyes and ICG with different excitations and long-pass filters. ([LZ dyes] = [ICG] = 10 μM). The bars represent mean ± s.d. derived from n = 3 independent vials. Source data are provided as a Source Data file.

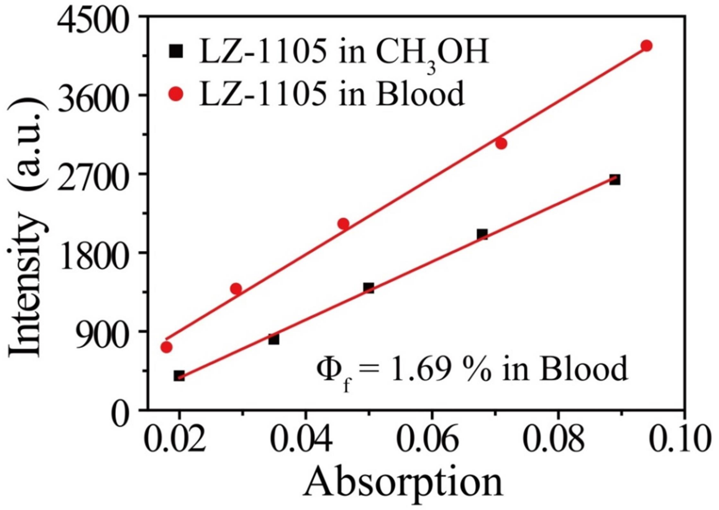

Supplementary Figure 7. Quantum yield of LZ-1105 in blood. The plot of fluorescence intensity of LZ-1105 in CH_3OH and blood at five different concentrations used to calculate the quantum yield in blood by comparing the slopes of the linear fits. The fluorescence quantum yield of LZ-1105 in blood was 1.69%. Source data are provided as a Source Data file.

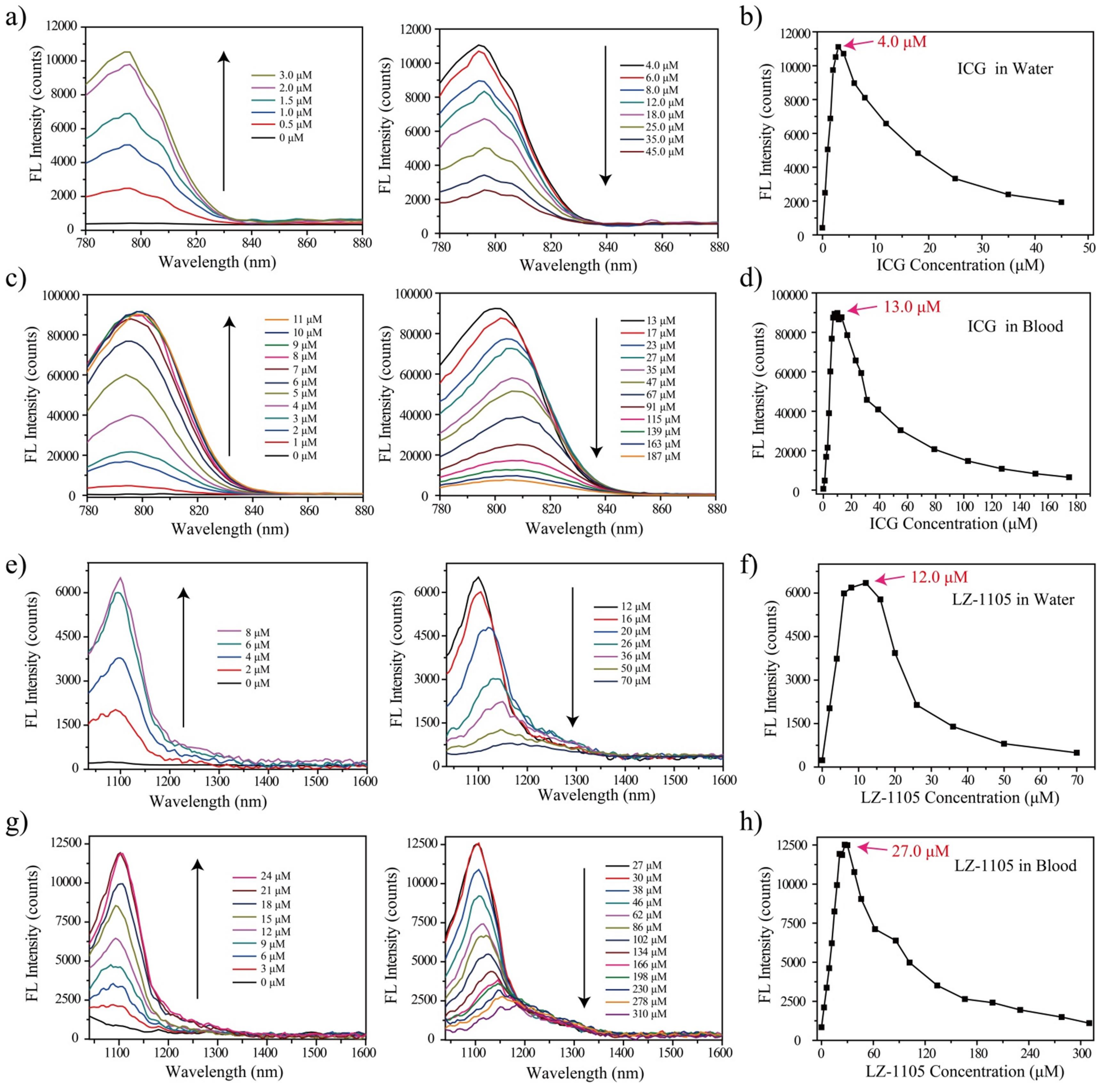

Supplementary Figure 8. Fluorescence spectra of ICG and LZ-1105 at various in different media. Fluorescence spectra of ICG and LZ-1105 at various concentration in water (ICG (**a**) and LZ-1105(**e**)) and blood (ICG (**c**) and LZ-1105 (**g**)). Fluorescence intensity at 794 nm for ICG in water (**b**) and in blood (**d**). Fluorescence intensity at 1105 nm for LZ-1105 in water (**f**) and in blood (**h**) as a function of incubation time.

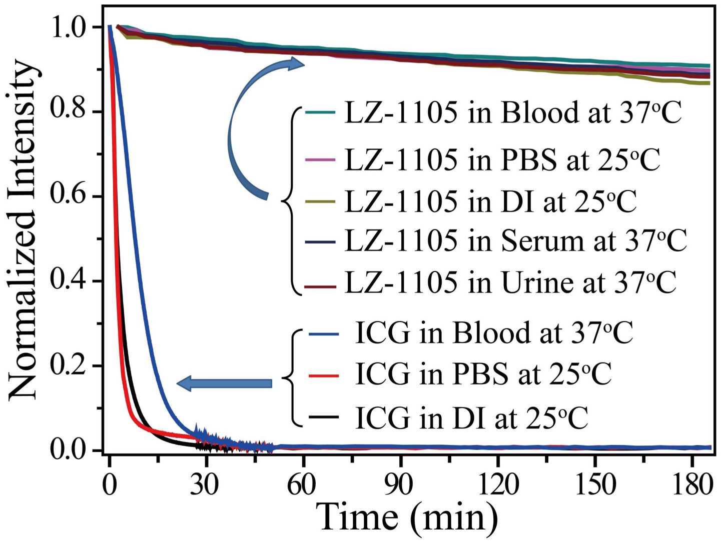

Supplementary Figure 9. Photostability of LZ-1105 and ICG. LZ-1105 and ICG in a variety of biological media under continuous 1064 nm and 808 nm lasers excitation exposure for 180 min (330 mW cm^{-2}), respectively. Source data are provided as a Source Data file.

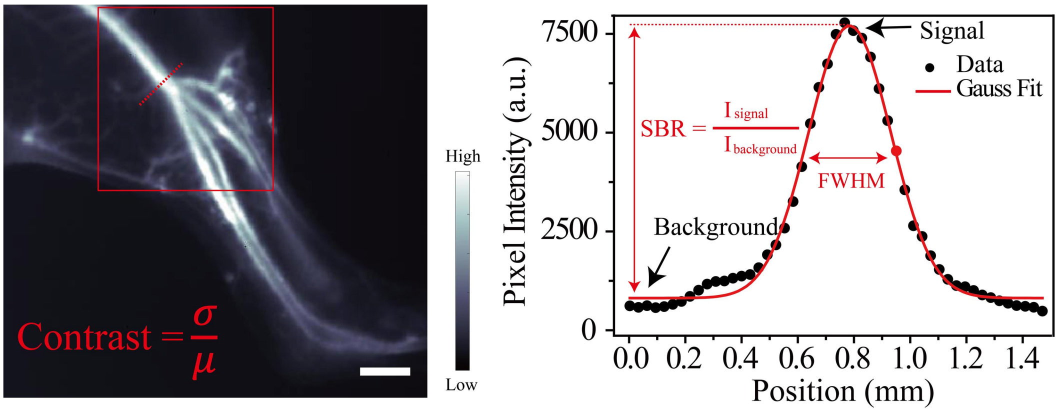

Supplementary Figure 10. Measurement method of signal-to background ratio, resolution and contrast in NIR-II bioimages. Scale bar represents 4 mm.

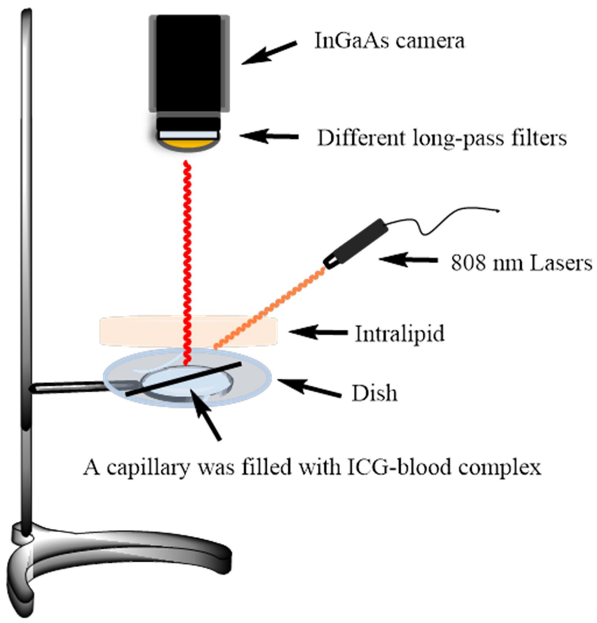

Supplementary Figure 11. The experimental setup illustration for ICG-blood complex imaging under various penetration depth through Intralipid.

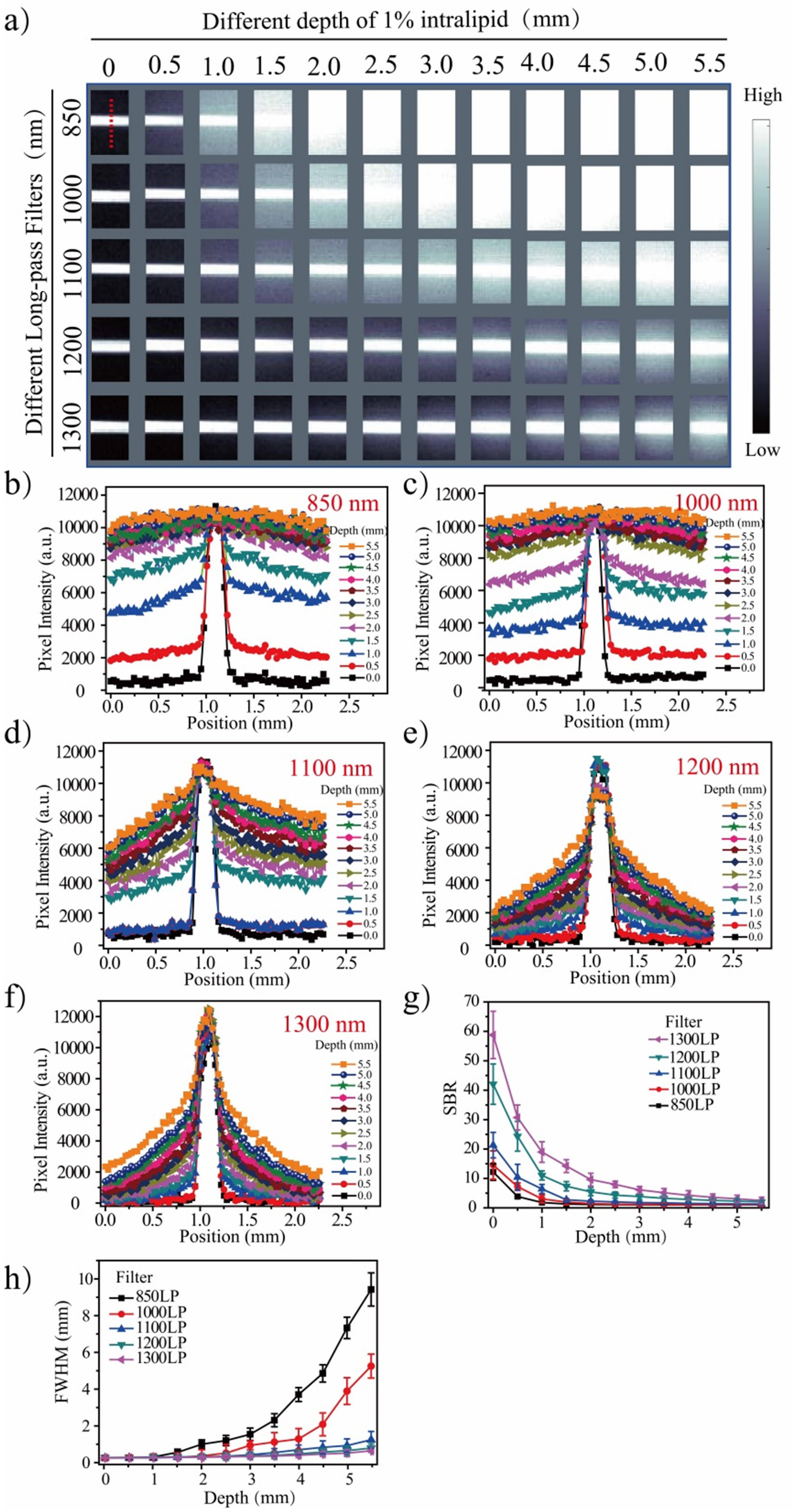
a)
Different depth of 1% intralipid (mm)
0 0.5 1.0 1.5 2.0 2.5 3.0 3.5 4.0 4.5 5.0 5.5
Different Long-pass Filters (nm)
850
1000
1100
1200
1300
High
Low
b)
850 nm
c)
1000 nm
d)
1100 nm
e)
1200 nm
f)
1300 nm
Pixel Intensity (a.u.)
Position (mm)
Depth (mm)
g)
SBR
Depth (mm)
Filter
1300LP
1200LP
1100LP
1000LP
850LP
h)
FWHM (mm)
Depth (mm)
Filter
850LP
1000LP
1100LP
1200LP
1300LP

Supplementary Figure 12. Deep penetration of ICG-filled single capillary in mimic tissue. **a** Fluorescence images of capillaries filled with ICG in blood ([ICG] = 10 μM) immersed in 1% Intralipid with varying depth. Imaging signals were collected with various long-pass filters under 808 excitation. **b-f** Profiles measured at same position in the capillary in various long-pass group at different depth. **g** Measured SBR of capillary images as a function of depth. The bars represent mean ± s.d., derived from n=3 line profiles measured at different positions in the capillary images. **h** Wavelength-dependent FWHM of cross-sectional profiles in capillary images as a function of depth. The bars represent mean ± s.d., derived from the uncertainty in the Gaussian fitting of feature width. Source data underlying b-h are provided as a Source Data file.

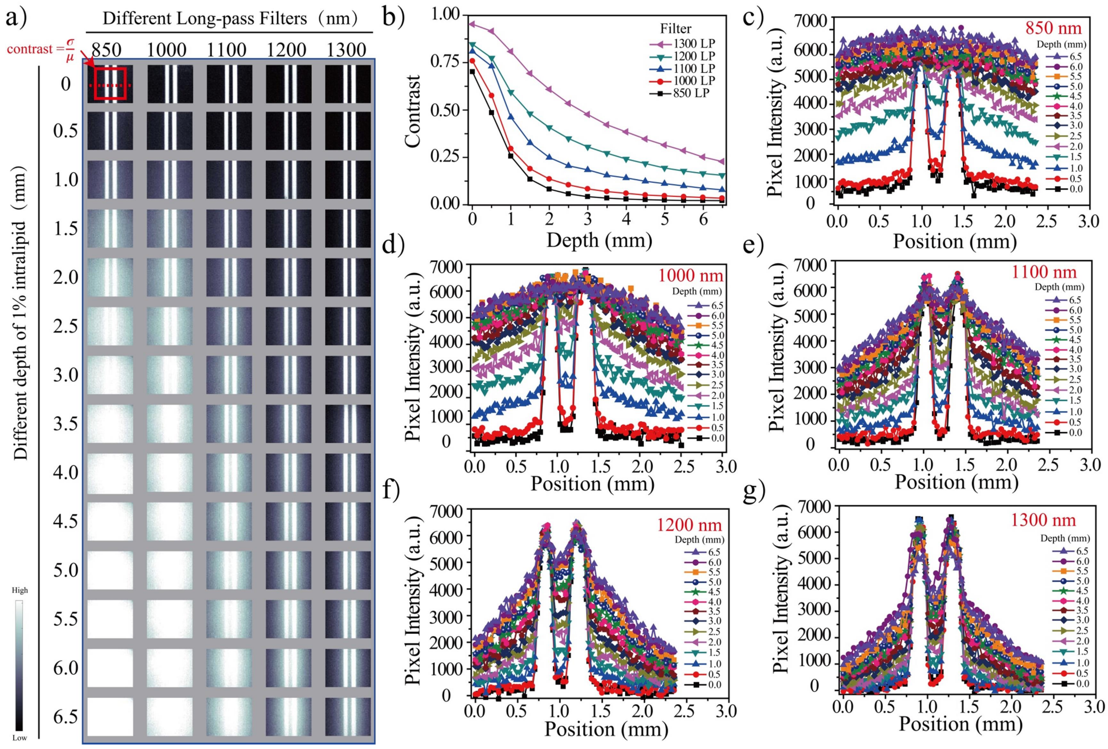

Supplementary Figure 13. Deep penetration of ICG-filled double capillaries in mimic tissue. **a** Fluorescence images of capillaries filled with ICG in blood ([ICG] = 10 μM) immersed in 1% Intralipid with varying depth. Imaging signals were collected with various long-pass filters under 808 excitation. **b** Wavelength-dependent contrast of capillary images as a function of depth. **c-g** Profiles measured at same position in the capillary in various long-pass groups at different depth. Source data underlying b-g are provided as a Source Data file.

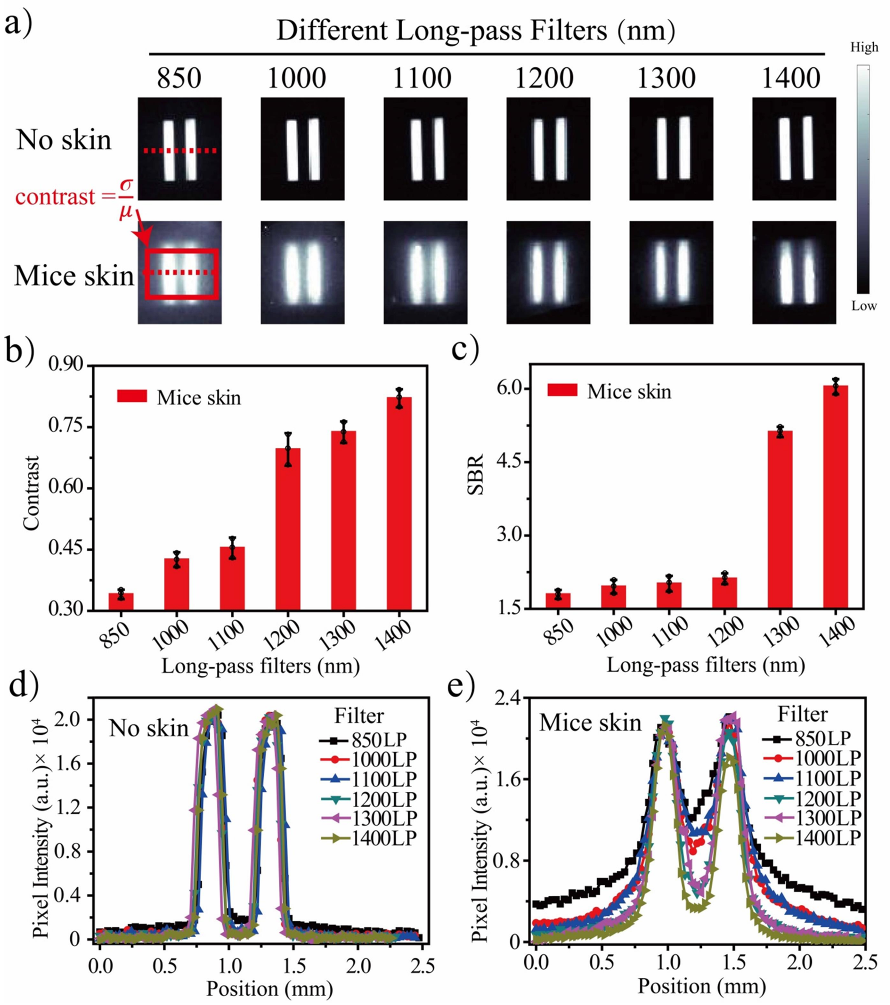

Supplementary Figure 14. Deep penetration of ICG-filled single capillary in mice skin. **a** Fluorescence images of capillaries filled with ICG in blood ([ICG] = 10 μM) immersed in mice skin. Imaging signals were collected with various long-pass filters under 808 excitation. Contrast was calculated in the red box. **b** Wavelength-dependent contrast of capillary images with or without mice skin. The bars represent mean ± s.d. derived from n=3 replicated measurements of every pixel at different positions in the capillary images. **c** Wavelength-dependent SBR of capillary images with or without mice skin. The bars represent mean ± s.d. derived from n=3 line profiles measured at different positions in the capillary images. **d-e** Profiles measured along the red dashed line in the capillary in various long-pass group with or without mice skin. Source data underlying b-e are provided as a Source Data file.

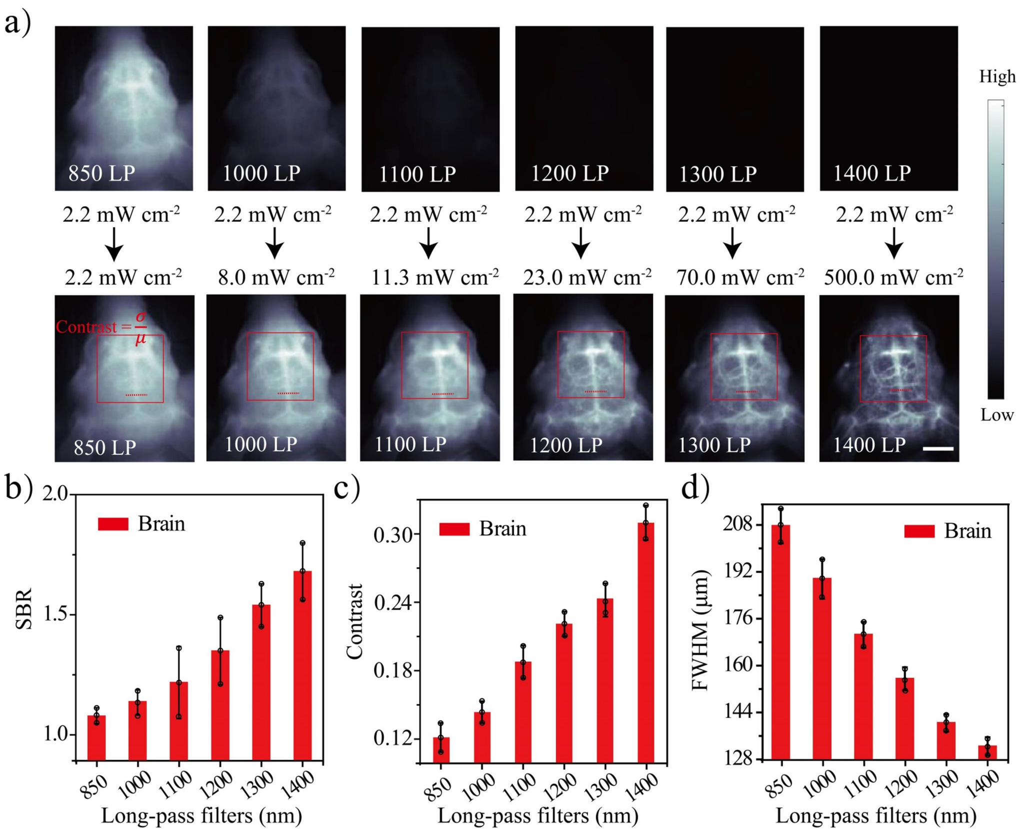

Supplementary Figure 15. NIR bioimaging of ICG injected mice brain with various long-pass filters. **a** NIR bioimaging of balb/c nude mice brain with various long-pass filters by ICG administration (up row: images were acquired under 808 nm excitation with same power density; bottom row: images were acquired under 808 nm excitation with different power density to obtain the same signal intensity, contrast was annotated as red letters). **b-d** SBR (**b**), contrast (**c**) and FWHM (**d**) of cross-sectional profiles in balb/c nude mice brain images with various long-pass filters. Data point with its error bar stands for mean ± s.d. derived from n=3 biologically independent mice. Scale bar represents 4 mm. Source data underlying b-d are provided as a Source Data file.

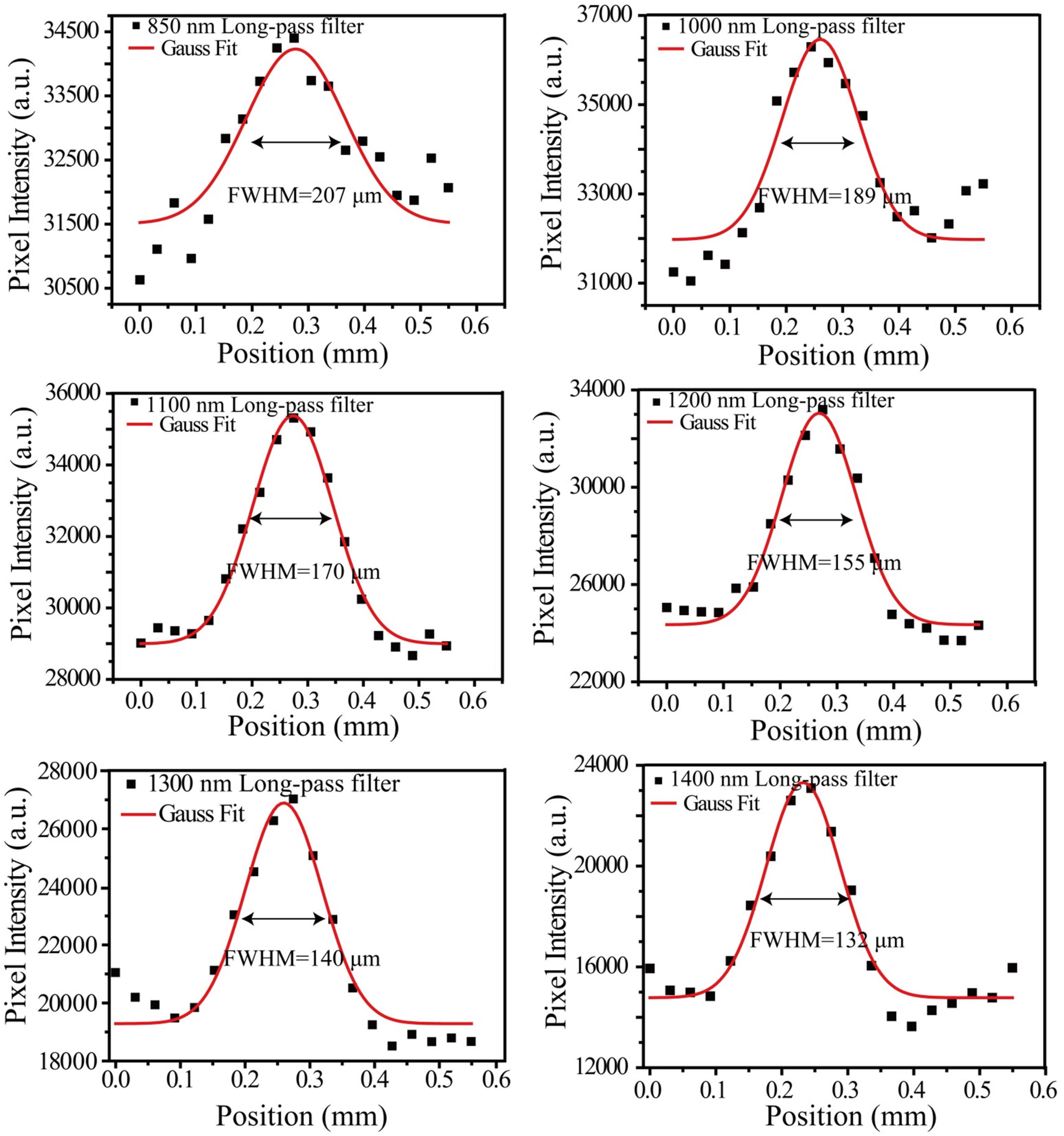

Supplementary Figure 16. FWHM of ICG injected mice brain obtained by various long-pass filters. The fluorescence intensity profiles (dots) and Gaussian fit (lines) along the red-dashed line in brain with various long-pass filters. Source data are provided as a Source Data file.

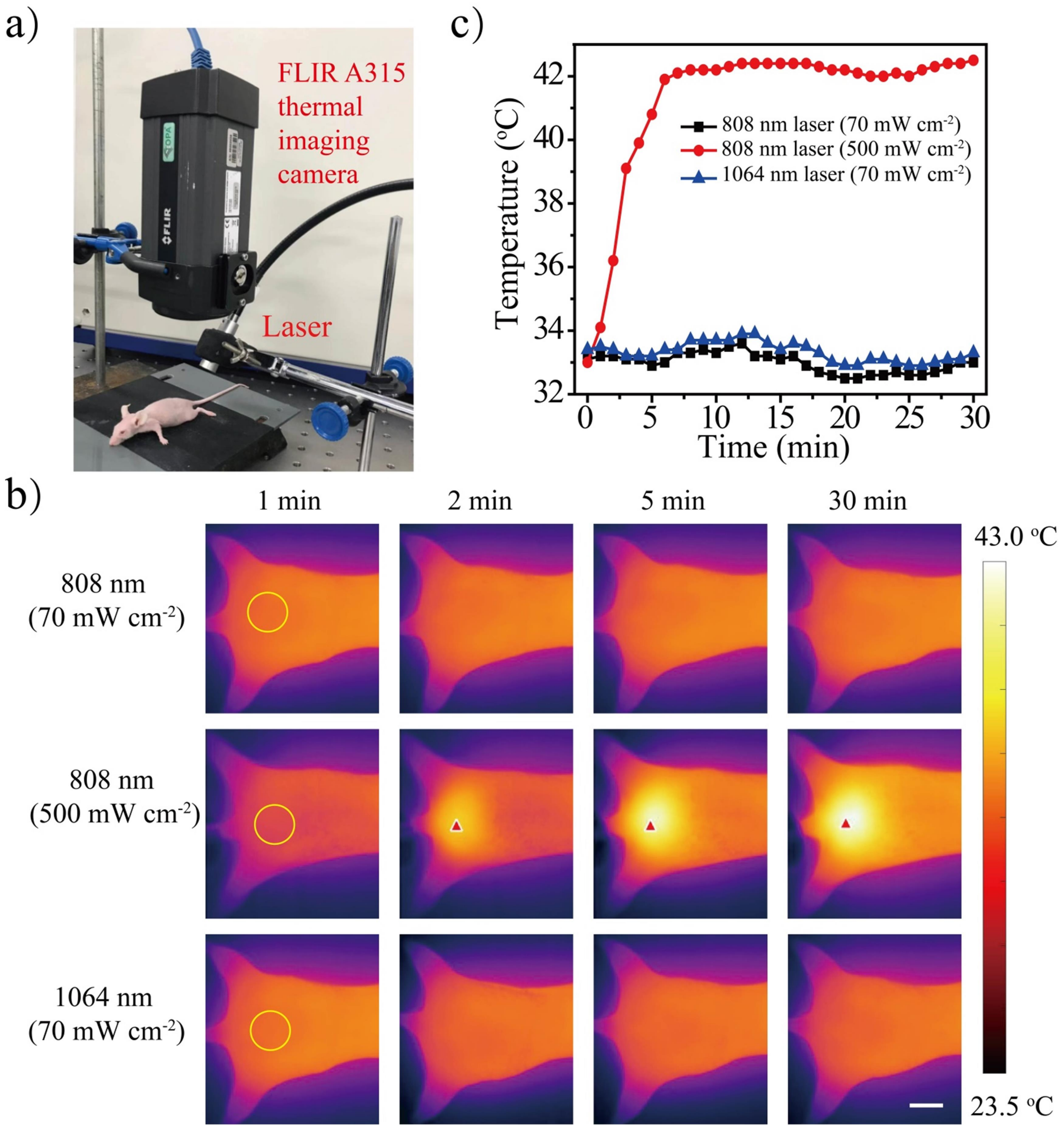

Supplementary Figure 17. Thermal effect experiments. Thermal effect experiments of 808 nm and 1064 nm lasers (70 or 500 mW cm^{-2}) irradiation on balb/c nude mice back using FLIR A315 thermal imaging camera. **a** Photograph of the experimental setup. **b** Photothermal images of mice back with different laser working power density for different irradiation time. The radiation area was shown with yellow circle. n=3 independent mice experiments. **c** Thermal effect for mice skin during 30 min irradiation of laser as a function of time. Scale bar represents 3 mm. Source data underlying c are provided as a Source Data file.

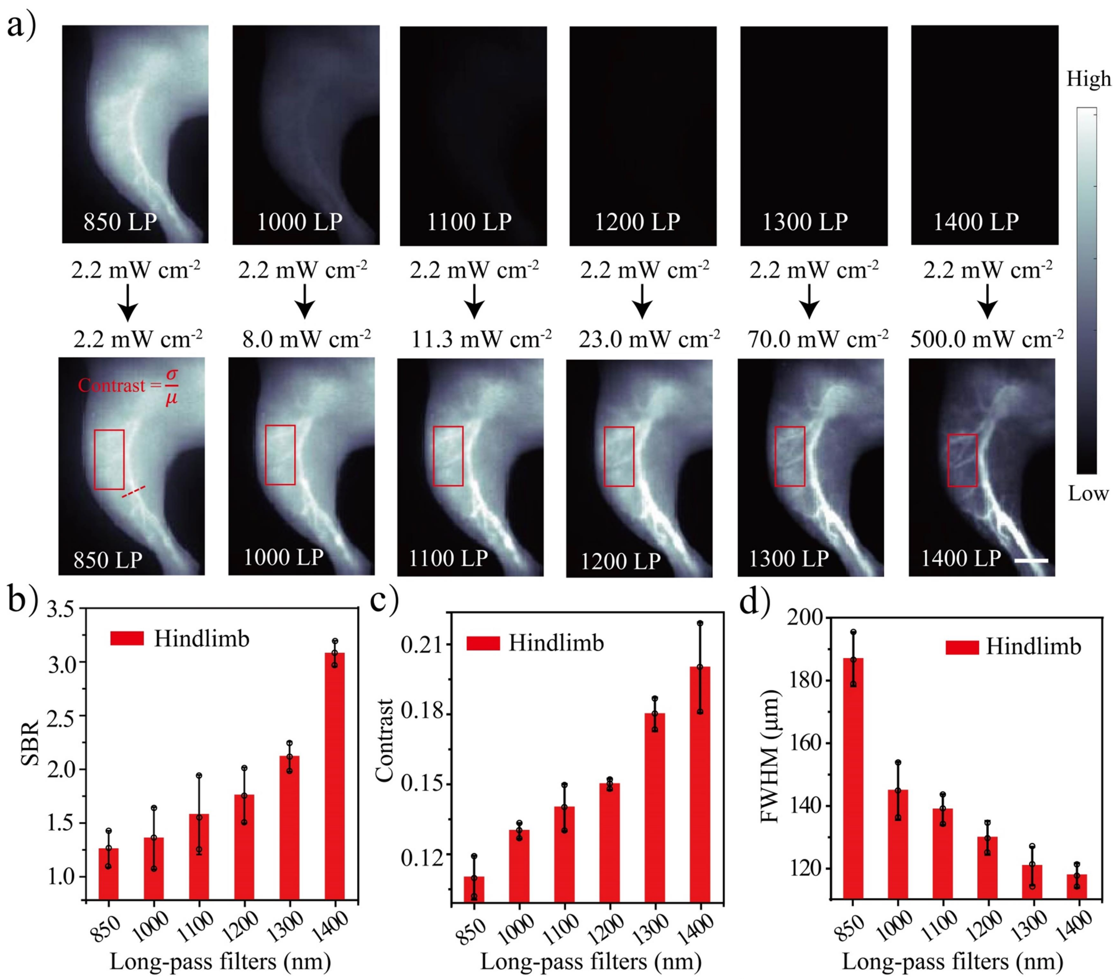

Supplementary Figure 18. NIR bioimaging of ICG injected mice hindlimbs with various long-pass filters. **a** NIR bioimaging of balb/c nude mice hindlimb with various long-pass filters by ICG administration (up row: images were acquired under 808 nm excitation with same power density; bottom row: images were acquired under 808 nm excitation with different power density to obtain the same signal intensity, contrast was annotated as red letters). Contrast was calculated in the red box. **b-d** SBR (**b**), contrast (**c**) and FWHM (**d**) of cross-sectional profiles in balb/c nude mice hindlimb images with various long-pass filters. Data point with its error bar stands for mean ± s.d. derived from n=3 biologically independent mice. Scale bar represents 4 mm. Source data underlying b-d are provided as a Source Data file.

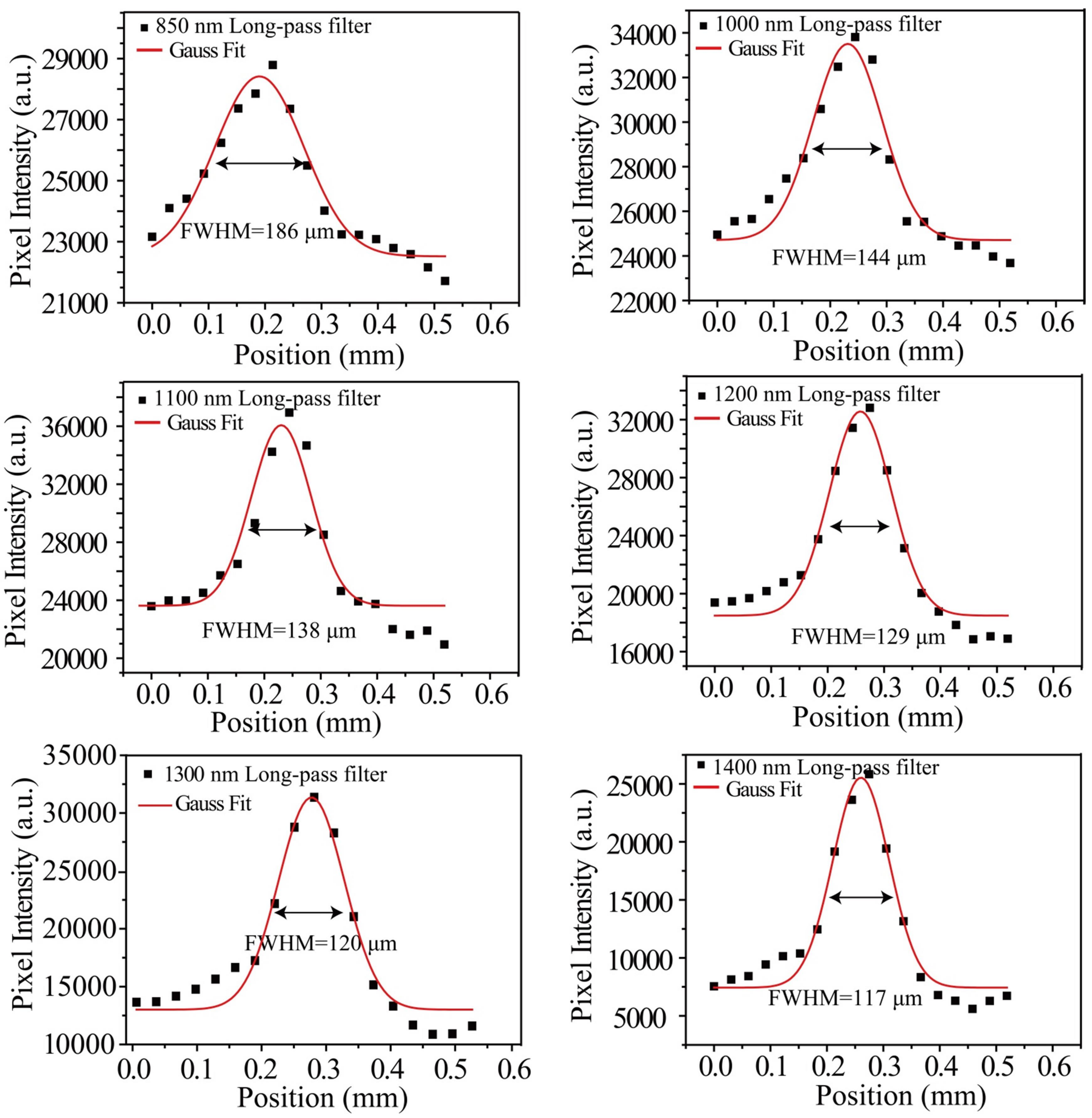

Supplementary Figure 19. FWHM of ICG injected mice hindlimb obtained by various long-pass filters. The fluorescence intensity profiles (dots) and Gaussian fit (lines) along the red-dashed line in hindlimb with various long-pass filters. Source data are provided as a Source Data file.

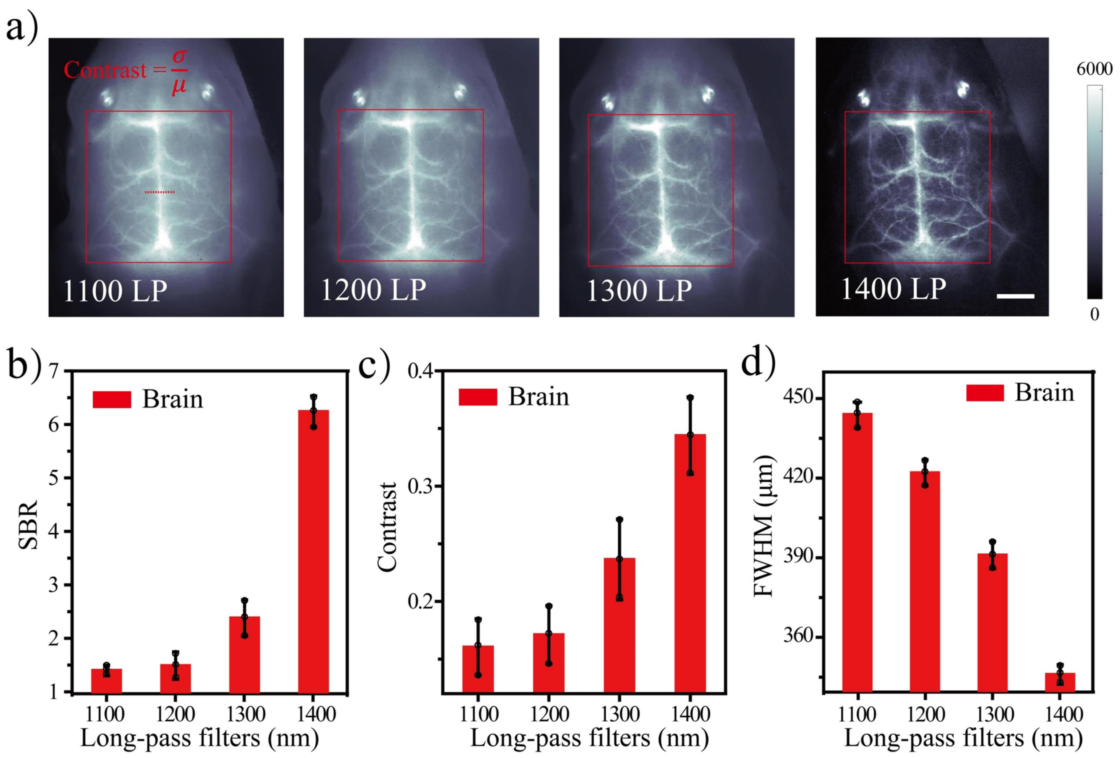

Supplementary Figure 20. NIR-II bioimaging of LZ-1105 injected mice brain with various long-pass filters. **a** NIR bioimaging of balb/c nude mice brain with various long-pass filters by LZ-1105 administration under 1064 nm excitation. Contrast was calculated in the red box. **b** SBR of balb/c nude mice brain imaging with various long-pass filters. **c** Contrast of brain vessels acquired by various long-pass filters. **d** FWHM of Gaussian fitted fluorescence intensity profiles of brain vessels acquired by various long-pass filters. Data point with its error bar stands for mean ± s.d. derived from n=3 biologically independent mice. Scale bar represents 4 mm. Source data underlying b-d are provided as a Source Data file.

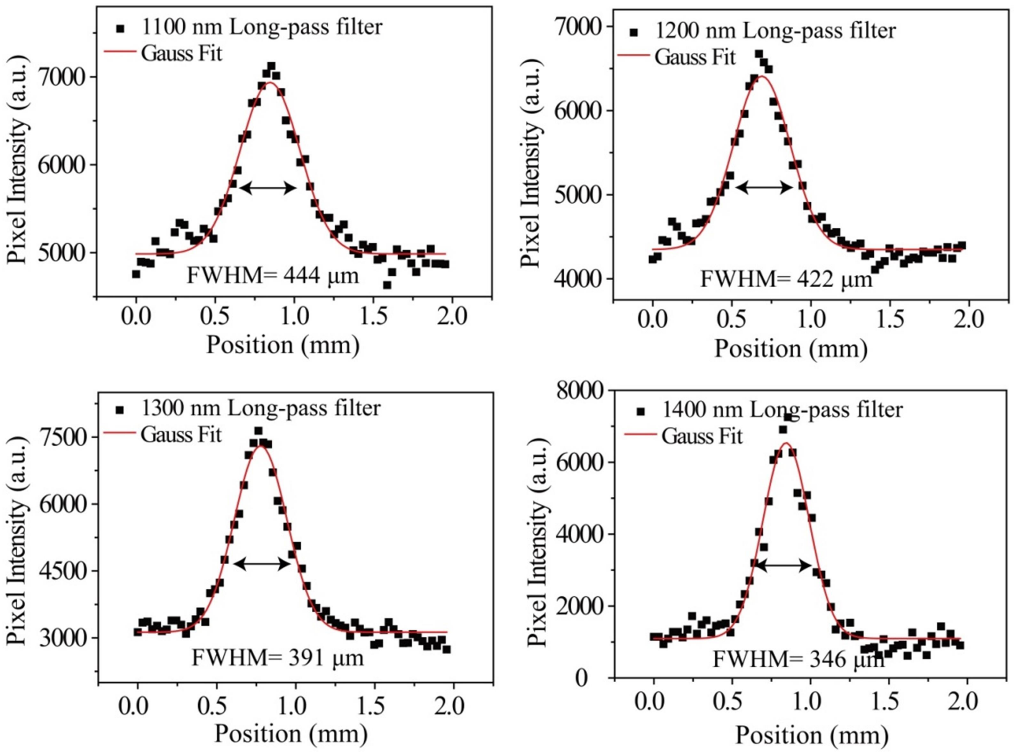

Supplementary Figure 21. FWHM of LZ-1105 injected mice brain obtained by various long-pass filters. The fluorescence intensity profiles (dots) and Gaussian fit (lines) along the red-dashed line in brain with various long-pass filters. Source data are provided as a Source Data file.

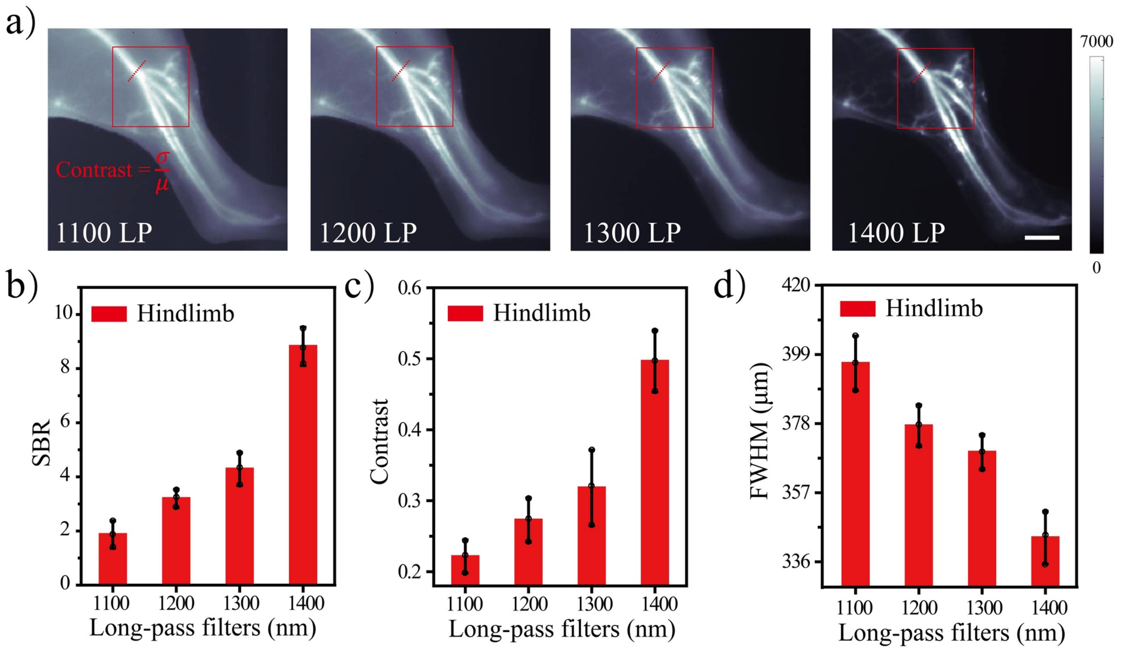

Supplementary Figure 22. NIR-II bioimaging of LZ-1105 injected mice hindlimbs with various long-pass filters. **a** NIR bioimaging of balb/c nude mice hindlimb with various long-pass filters by LZ-1105 administration under 1064 nm excitation. Contrast was calculated in the red box. **b** SBR of balb/c nude mice hindlimb imaging with various long-pass filters. **c** Contrast of hindlimb vessels acquired by various long-pass filters. **d** FWHM of Gaussian fitted fluorescence intensity profiles of hindlimb vessels acquired by various long-pass filters. Data point with its error bar stands for mean ± s.d. derived from n=3 biologically independent mice. Scale bar represents 4 mm. Source data underlying b-d are provided as a Source Data file.

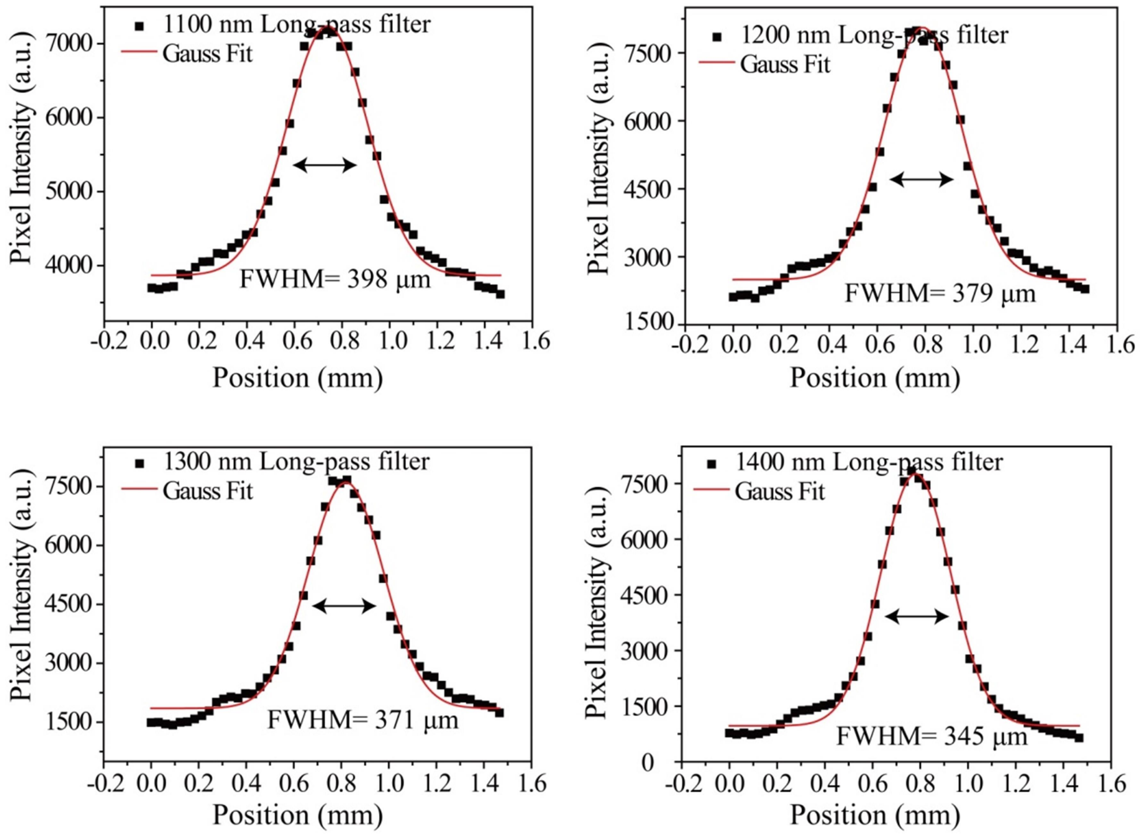

Supplementary Figure 23. FWHM of LZ-1105 injected mice hindlimb obtained by various long-pass filters. The fluorescence intensity profiles (dots) and Gaussian fit (lines) along the red-dashed line in hindlimb with various long-pass filters. Source data are provided as a Source Data file.

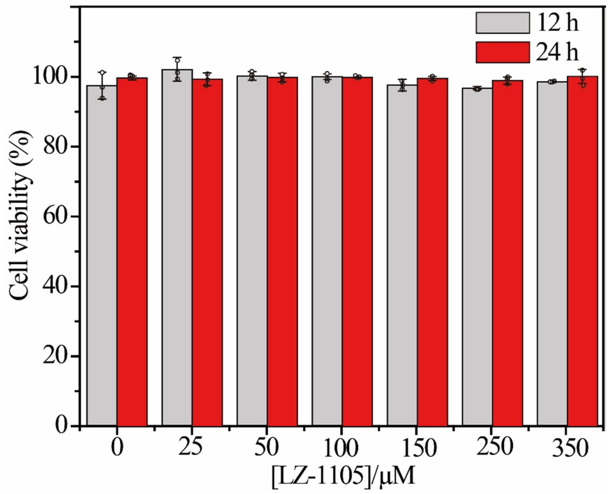

Supplementary Figure 24. Potential cytotoxicity of LZ-1105. Cell viability of human umbilical vein endothelial cells (HUVEC) after 12 h and 24 h incubation with LZ-1105 under different concentrations. The cell viabilities were above 95 % even with concentration of 350 μM. Error bars represent standard deviation. All samples were done in triplicate and the experiment was replicated three times. Source data are provided as a Source Data file.

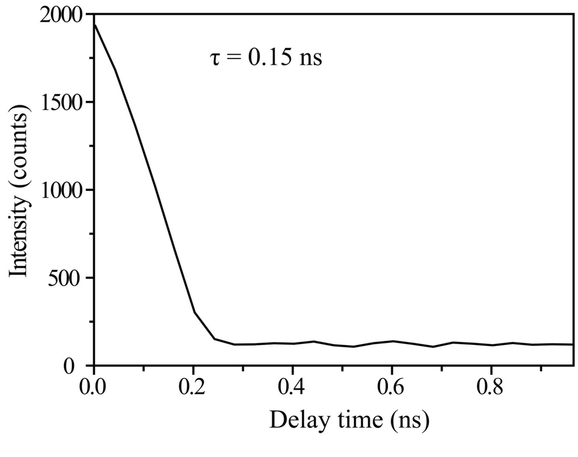

Supplementary Figure 25. Lifetime of LZ-1105 in water phase. Luminescence decay curve measured at 1105 nm under the excitation of 1064 nm. Source data are provided as a Source Data file.

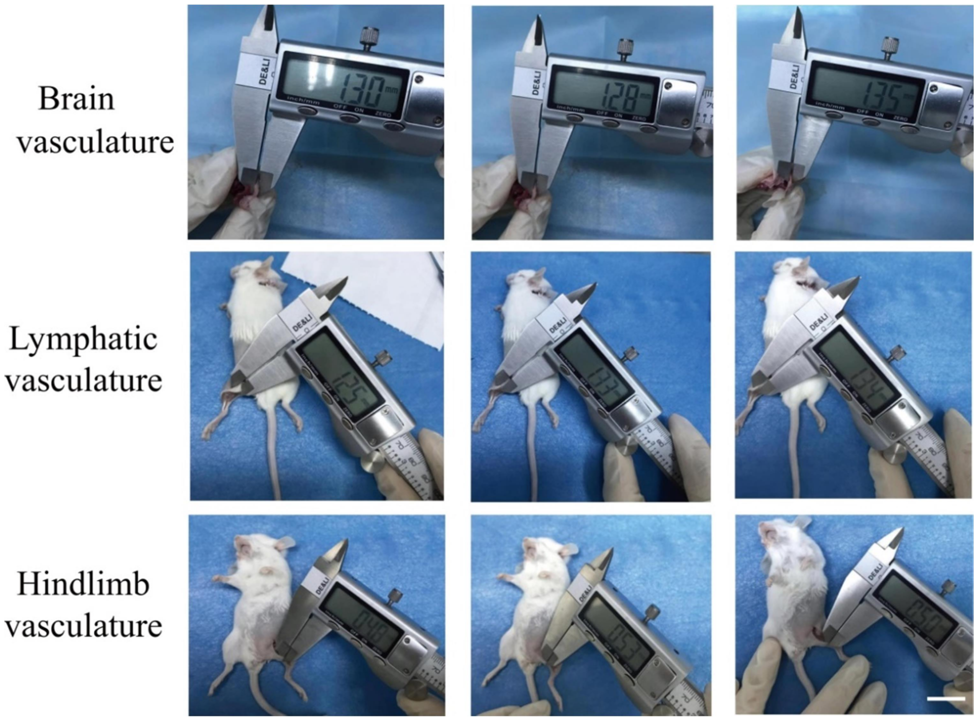

Supplementary Figure 26. Penetration depth of brain, lymphatic and hindlimb vasculatures. Precise penetration depth was measured by vernier caliper after sacrificing the imaging mice. The average thickness were 1.31 mm, 1.31 mm and 0.50 mm, respectively. Scale bar represent 2 cm.

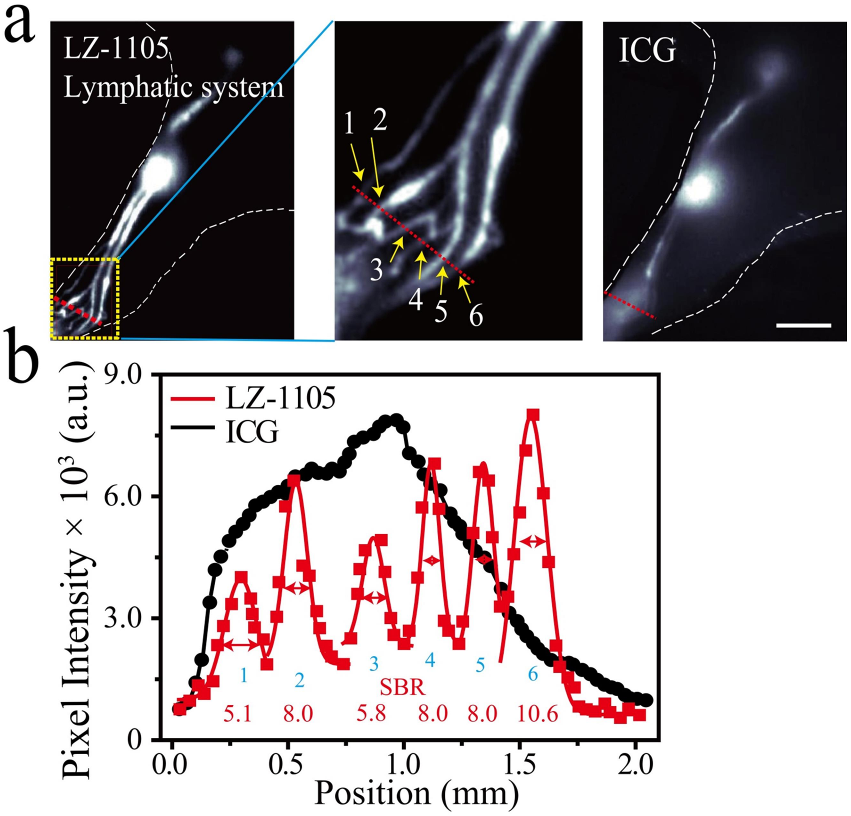

Supplementary Figure 27. NIR-II bioimaging of lymphatic system in ICG and LZ-1105 injected mice. **a** Non-invasive NIR-II fluorescence images of lymphatic system in shaved ICR mice i.v. injected with LZ-1105 (1400 long-pass filter, λ_{ex}=1064 nm, 300 ms) or ICG (1300 long-pass filter, λ_{ex}=808 nm, 300 ms). n=3 independent mice experiments. **b** The fluorescence intensity profiles (dots) and Gaussian fit (lines) along the red-dashed line in lymphatic system. Scale bar represents 4 mm. Source data underlying b are provided as a Source Data file.

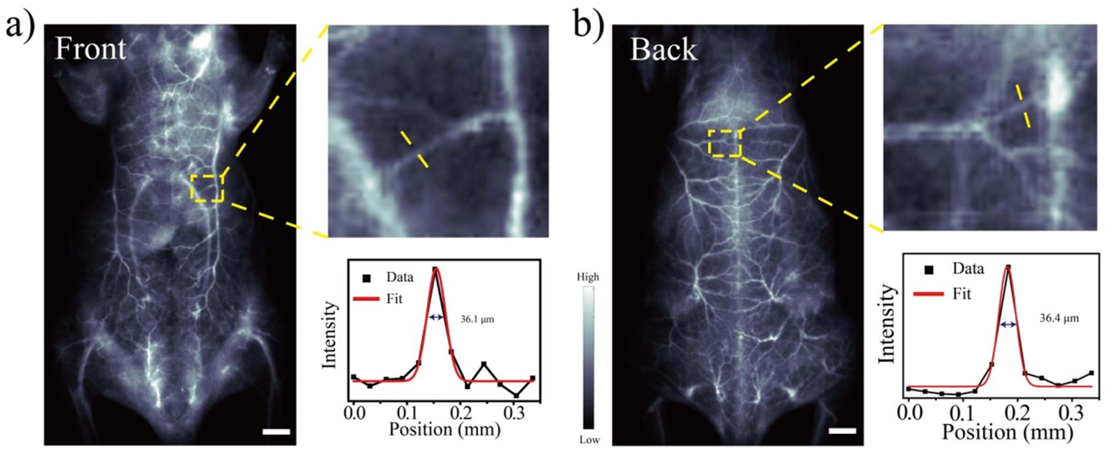

Supplementary Figure 28. Whole body NIR-II fluorescence imaging with LZ-1105 at front (**a**) and back (**b**) of mice. The whole body NIR-II fluorescence imaging of mice after i.v. injection of LZ-1105. (λ_{ex} = 1064 nm, 1400 nm long-pass filter), the smallest measurable vessel exhibited a Gaussian-fit diameter of only 36.1 μm and 36.4 μm on the front and back side, respectively, illustrating the high resolution achieved by LZ-1105 administrated *in vivo* NIR-II imaging. Scale bars represent 7 mm. Source data are provided as a Source Data file.

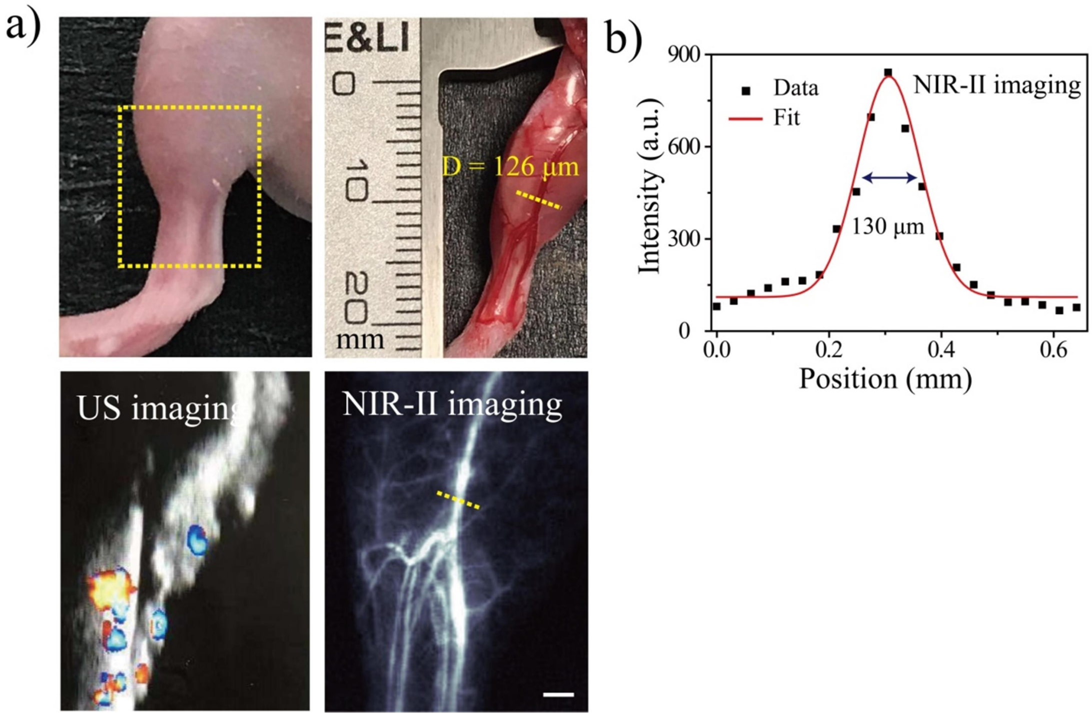

Supplementary Figure 29. The digital photograph, Ultrasound imaging and NIR-II imaging of hindlimb in LZ-1105 injected mice. **a** The digital camera photograph of left leg in mouse with or without skin, Ultrasound imaging and NIR-II fluorescence imaging (λ_{ex} =1064 nm, 1400 nm long-pass filter, 300 ms) of the same area in hindlimb blood vessels. **b** The fluorescence intensity profiles (dots) and Gaussian fit (lines) along the yellow-dashed line in NIR-II imaging. Scale bar represents 3 mm. Source data underlying b are provided as a Source Data file.

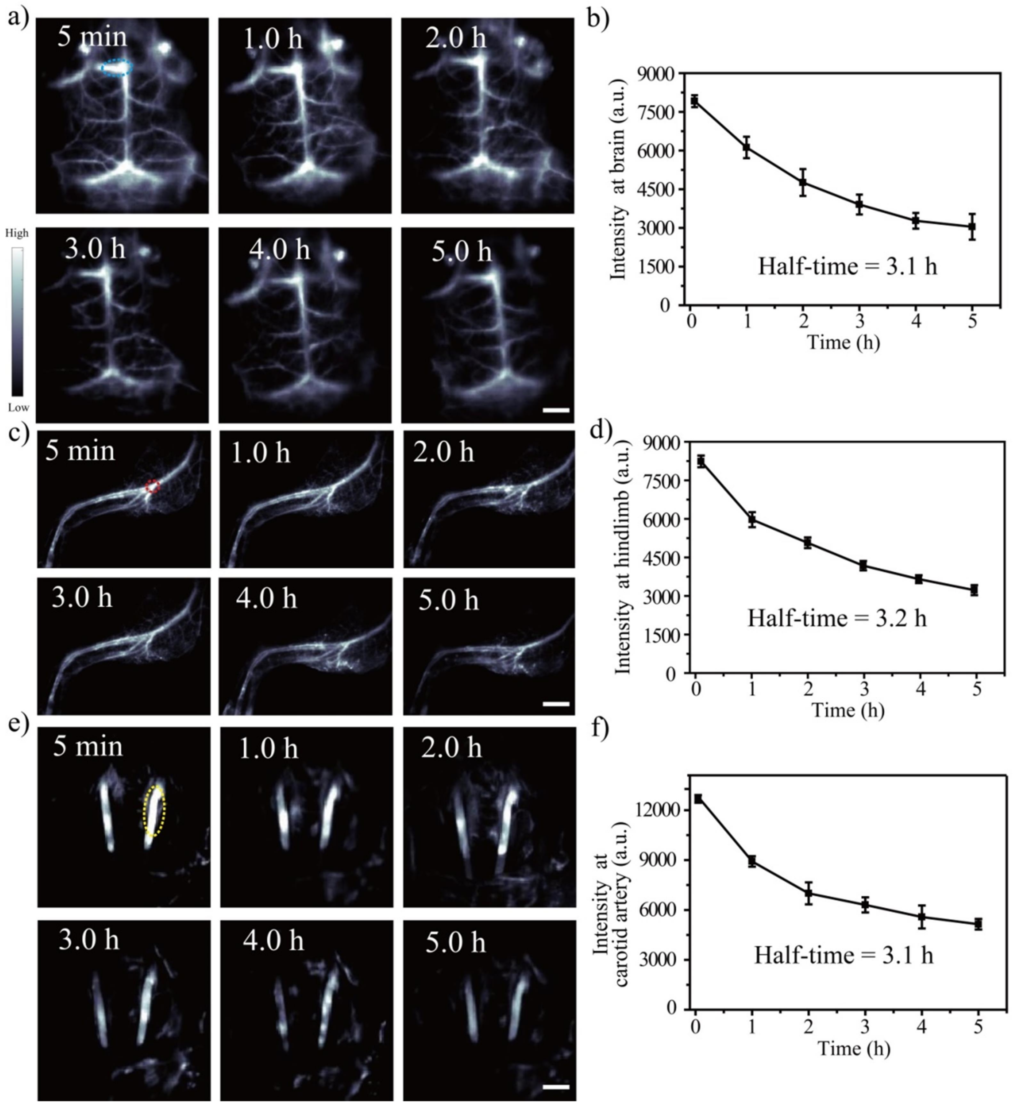

Supplementary Figure 30. In vivo NIR-II imaging of the mice injected with LZ-1105. **a, c, e.** NIR-II images of the mice brain (**a**), hindlimb (**c**) and carotid artery (**e**) as a function of time after i.v. injection of LZ-1105 (λ_{ex}=1064 nm, 1400 nm long-pass filter). **b, d, f.** Representative fluorescence signal intensity of brain (blue circle) (**b**), hindlimb (red circle) (**d**) and carotid artery (yellow circle) (**f**) in (**a, c, e**), blood circulation half-life time were measured as 3.1 h, 3.2 h and 3.1 h for brain, hindlimb and carotid artery, respectively, illustrating the long blood half-life time of LZ-1105. Data point with its error bar stands for mean ± s.d. derived from n=3 biologically independent mice. Scale bars in a, c and e represent 2, 1 and 3 mm, respectively. Source data underlying b, d and f are provided as a Source Data file.

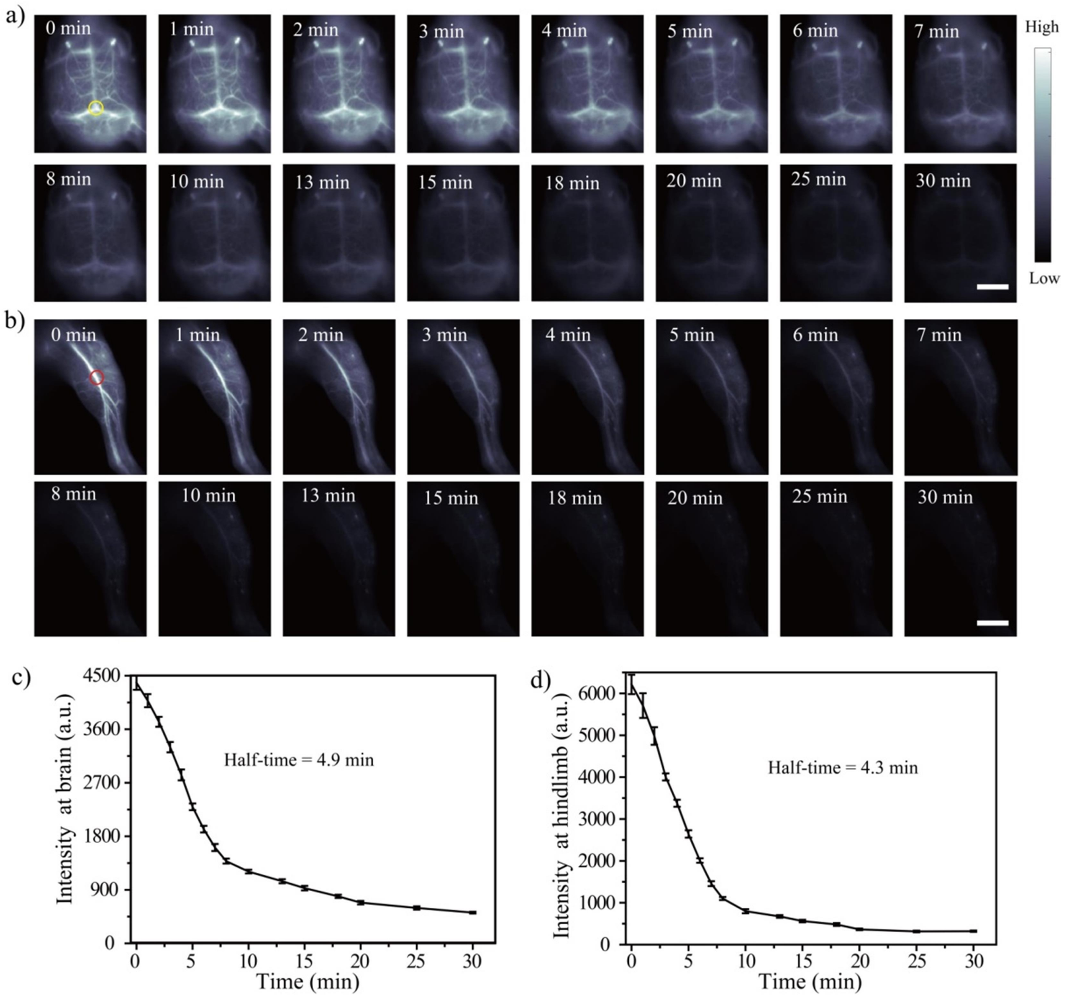

Supplementary Figure 31. In vivo NIR-II imaging of the mice injected with ICG. **a, b.** NIR-II images of the mice brain (**a**) and hindlimb (**b**) as a function of time after i.v. injection of ICG (λ_{ex}=808 nm, 1300 nm long-pass filter). **c,d.** Representative fluorescence signal intensity of brain (yellow circle) (**c**) and hindlimb (red circle) (**d**) in (**a**) and (**b**), blood circulation half-life time were measured as 4.9 min and 4.3 min for brain and hindlimb, respectively, illustrating the short blood half-life time of ICG. Data point with its error bar stands for mean ± s.d. derived from n=3 biologically independent mice. Scale bars in a and b represent 4 and 3 mm, respectively. Source data underlying c and d are provided as a Source Data file.

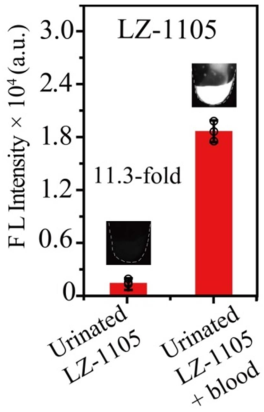

Supplementary Figure 32. Optical characterization of LZ-1105 in urine solvents. The fluorescence intensity of LZ-1105 in urine detected by the InGaAs camera (1064 nm excitation for LZ dyes, 30 mW cm^{-2}, 1400 nm long-pass filter, 300 ms). The bars represent mean ± s.d. derived from n = 3 biologically independent mice. Source data are provided as a Source Data file.

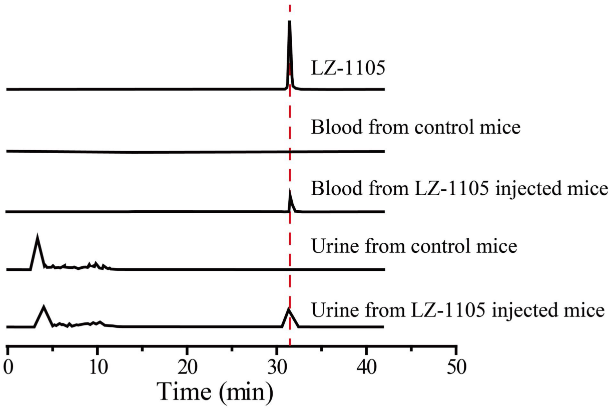

Supplementary Figure 33. In vivo stability of LZ-1105 measured by RP-HPLC. RP-HPLC results of LZ-1105, treated blood samples from control and LZ-1105 injected mice, treated urine samples from control and LZ-1105 injected mice. An elution peak of the treated urine sample at 31.5 min corresponding to free LZ-1105 was observed in RP-HPLC trace of the solution (Supplementary Figure 33). These results indicated the stability and renal clearance of LZ-1105 in vivo. Meanwhile, an elution peak at 31.5 min was observed in RP-HPLC trace of the treated blood sample, illustrating the good stability of LZ-1105 in blood.

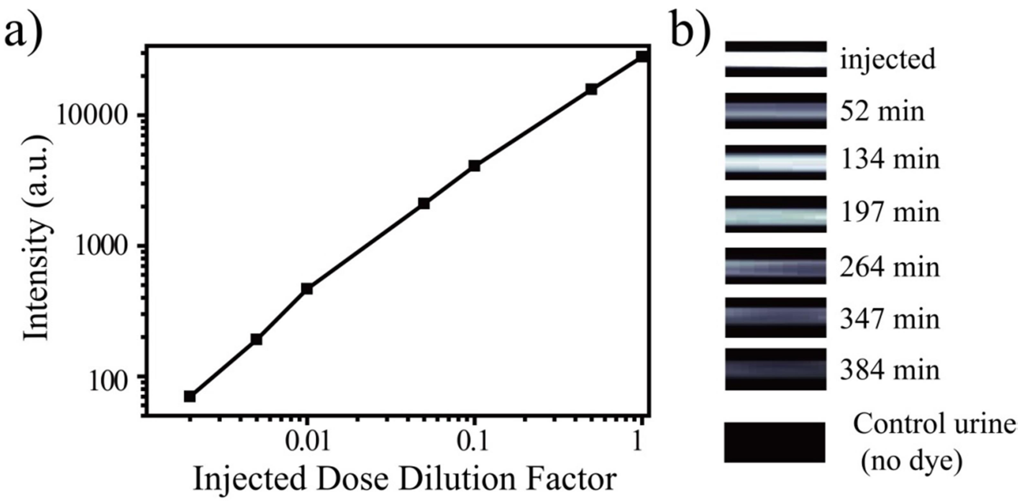

Supplementary Figure 34. Measurement of LZ-1105 content in urine. **a** The linearity of the fluorescence emission was verified by average fluorescence intensity of the injected LZ-1105 dose and then a series of dilutions. **b** NIR-II fluorescence images of capillary tubes (λ_{ex}=1064 nm, 1400 nm long-pass filter) corresponding to the injected dose of LZ-1105 and the urine samples collected without coercion. **c** Urine excretion of LZ-1105 as a function of time. Source data underlying a are provided as a Source Data file.

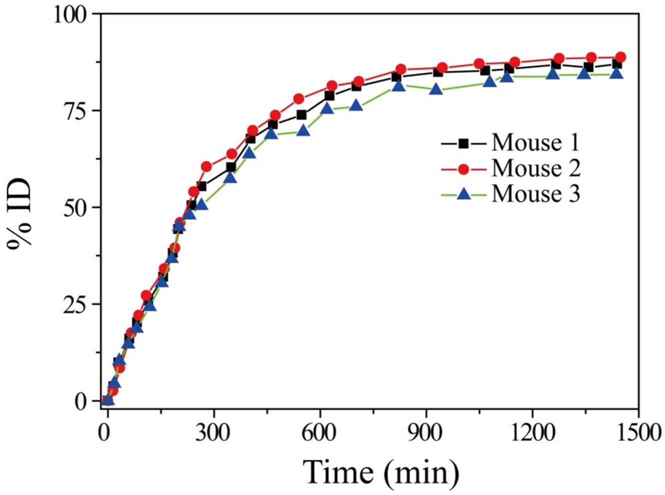

Supplementary Figure 35. LZ-1105 urine excretion from individual ICR mice. Urine excretion profiles from the 3 individual mice as a function of time. Source data are provided as a Source Data file.

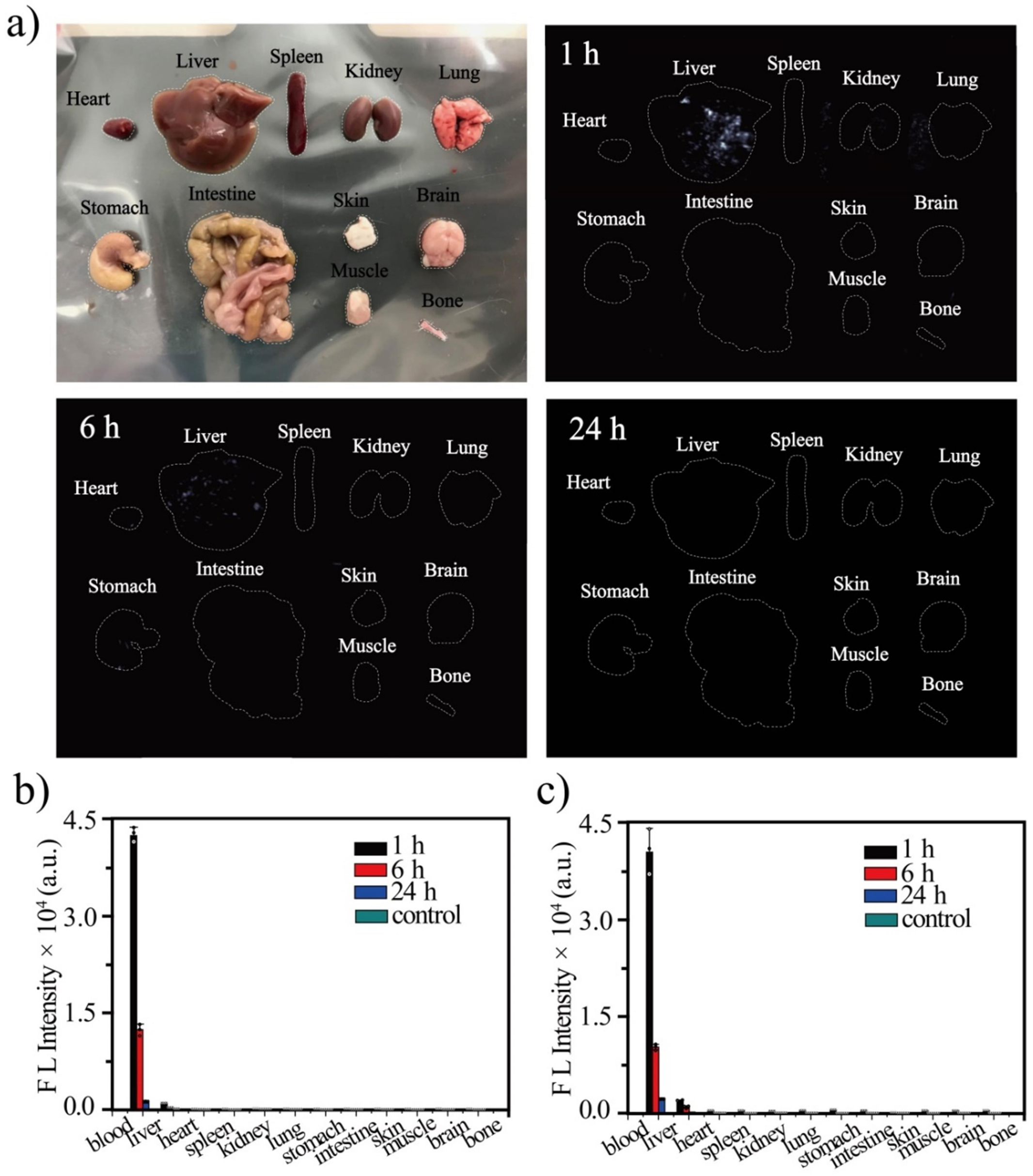

Supplementary Figure 36. LZ-1105 distribution in main organs and tissues. **a** Ex vivo images of main organs of LZ-1105 treated mice after 1 h, 6 h and 24 h p.i.. **b-c** Signal intensity of organs and tissues measured by InGaAs camera before (**b**) and after (**c**) homogenizing. The bars represent mean ± s.d. derived from n = 3 biologically independent mice. Source data underlying **b** and **c** are provided as a Source Data file.

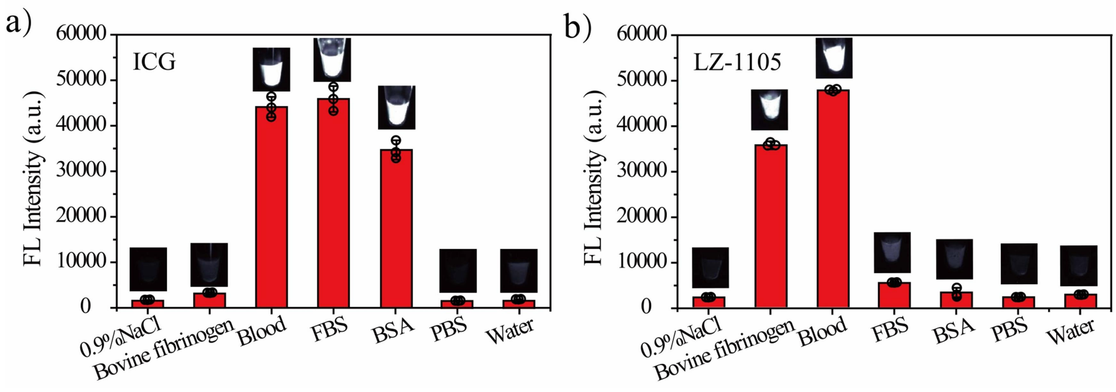

Supplementary Figure 37. Optical characterization of ICG and LZ-1105 in various media. The fluorescence intensity of ICG (**a**) and LZ-1105 (**b**) in mice blood detected by the InGaAs camera (808 nm excitation for ICG, 38 mW cm^{-2}, 1300 nm long-pass filter; 1064 nm excitation for LZ-1105, 30 mW cm^{-2}, 1400 nm long-pass filter). Insets: NIR-II fluorescence images of ICG and LZ-1105 with different excitations and long-pass filters. ([LZ-1105] = [ICG] = 10 μM). The bars represent mean ± s.d. derived from n = 3 independent vials. Source data are provided as a Source Data file.

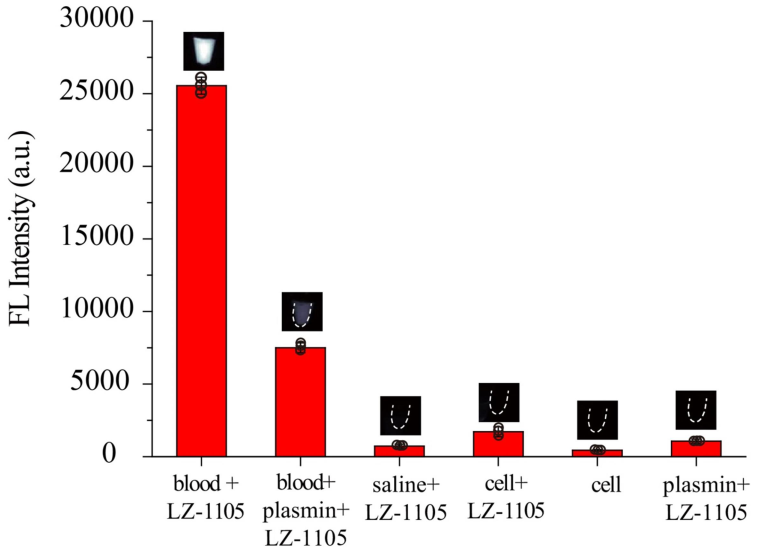

Supplementary Figure 38. Optical characterization of LZ-1105 in different media. The fluorescence intensity of LZ-1105 detected by the InGaAs camera in different media including fresh mice blood with or without plasmin, saline (0.9% NaCl solution), human umbilical vein endothelial cells (HUVEC) suspension and plasmin solution (1064 nm excitation, 30 mW cm^{-2}, 1400 nm long-pass filter). Insets: NIR-II fluorescence images of LZ-1105 in different media. ([LZ-1105] = 10 μM). The bars represent mean ± s.d. derived from n = 3 independent vials. Source data are provided as a Source Data file.

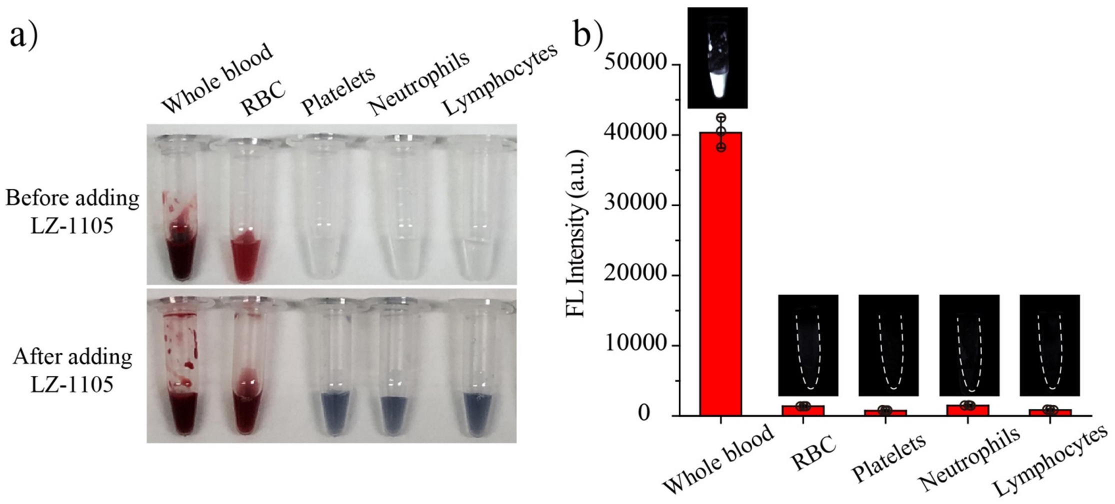

Supplementary Figure 39. Measurement of the interaction between whole blood or blood cells and LZ-1105. **a** White light photos of mouse whole blood and blood cells before and after adding LZ-1105. Blood cells were obtained from ICR mice by density gradient centrifugation. **b** NIR-II imaging results of LZ-1105 in mouse whole blood, RBC, platelets, neutrophils, and lymphocytes. (1064 nm excitation, 30 mW cm^{-2}, 1400 nm long-pass filter). Insets: NIR-II fluorescence images of LZ-1105 in different media. ([LZ-1105] = 10 μM). The bars represent mean ± s.d. derived from n = 3 independent vials. Source data underlying b are provided as a Source Data file.

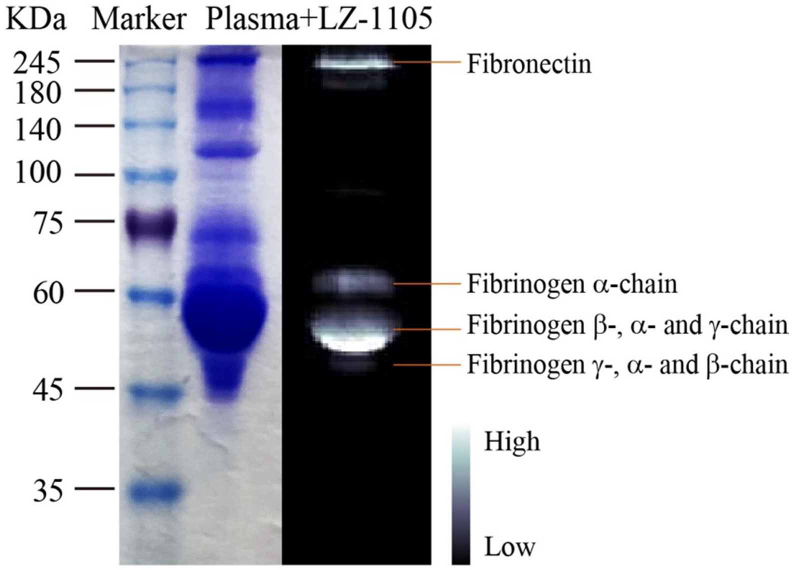

Supplementary Figure 40. Sodium dodecyl sulfate polyacrylamide gel electrophoresis (SDS-PAGE) results. Electrophoresis gel analysis of LZ-1105-plasma complexes and NIR-II imaging of the electrophoresis gel.[1] Concentration of LZ-1105: 10 μM. Power density of 1064 nm laser: 30 mW cm^{-2}. NIR-II images were acquired by 1400 long-pass filter. n=3 independent experiments.

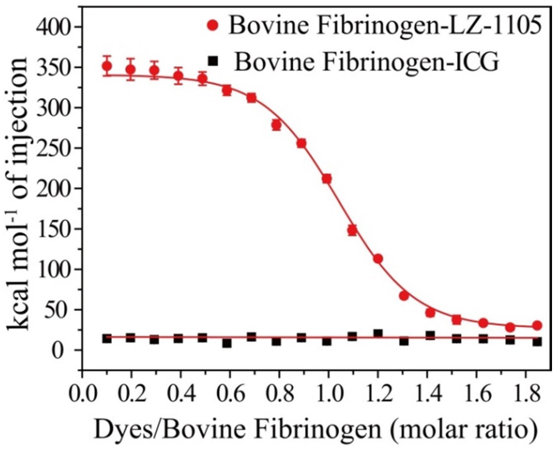

Supplementary Figure 41. Measurement of the LZ-1105 and ICG binding ability with bovine fibrinogen. Isothermal titration calorimetry (ITC) data titration results of titration of LZ-1105 (0.1 mM) or ICG (0.1 mM) into 0.01 mM bovine fibrinogen at 20°C in aqueous solution. The bars represent mean ± s.d. derived from n = 3 independent vials. Source data are provided as a Source Data file.

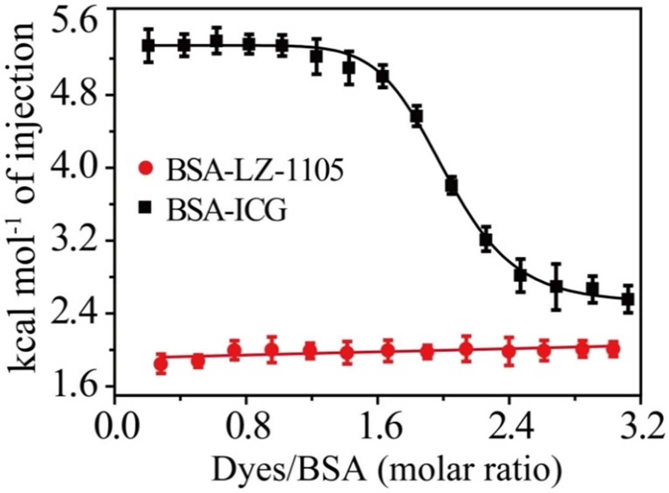

Supplementary Figure 42. Measurement of the LZ-1105 and ICG binding ability with bull serum albumin (BSA). Isothermal titration calorimetry (ITC) data titration results of titration of LZ-1105 (0.1 mM) or ICG (0.1 mM) into 0.01 mM BSA at 20°C in aqueous solution. The bars represent mean ± s.d. derived from n = 3 independent vials. Source data are provided as a Source Data file.

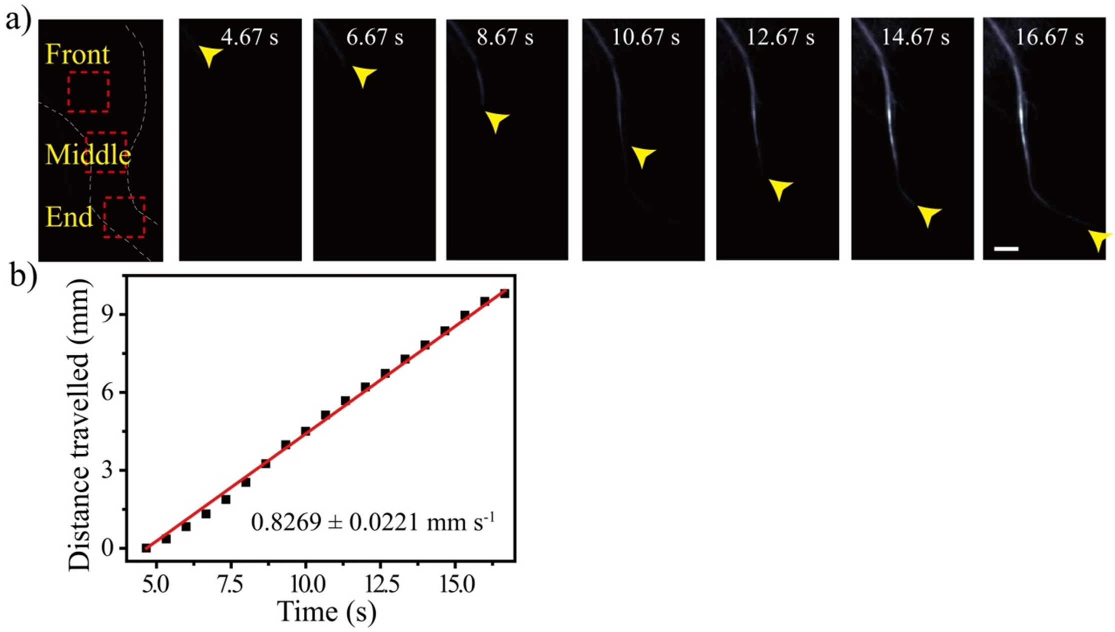

Supplementary Figure 43. Calculation of the ischemic reperfusion blood flow velocity (BFV) in mice hindlimb after clipping for 1 h. **a** NIR-II images of ischemic reperfusion at different time points after clipping for 1 h with LZ-1105 administration (λ_{ex} = 1064 nm, 1400 nm long-pass filter, 300 ms). Yellow arrows indicate the flow front. **b** The corresponding distance travelled by the flow front as a function of time. The slope of the function was calculated as BFV. When the clipping time was 1 h, the BFV in the front, middle and end site were comparable, illustrating the reperfusion in the ischemic hindlimb was fluent and fast. The slope of the function was calculated as blood flow velocity (BFV) and indicates mean ± s.d. derived from n=3 replicated measurements. Scale bar represents 4 mm. Source data underlying b are provided as a Source Data file.

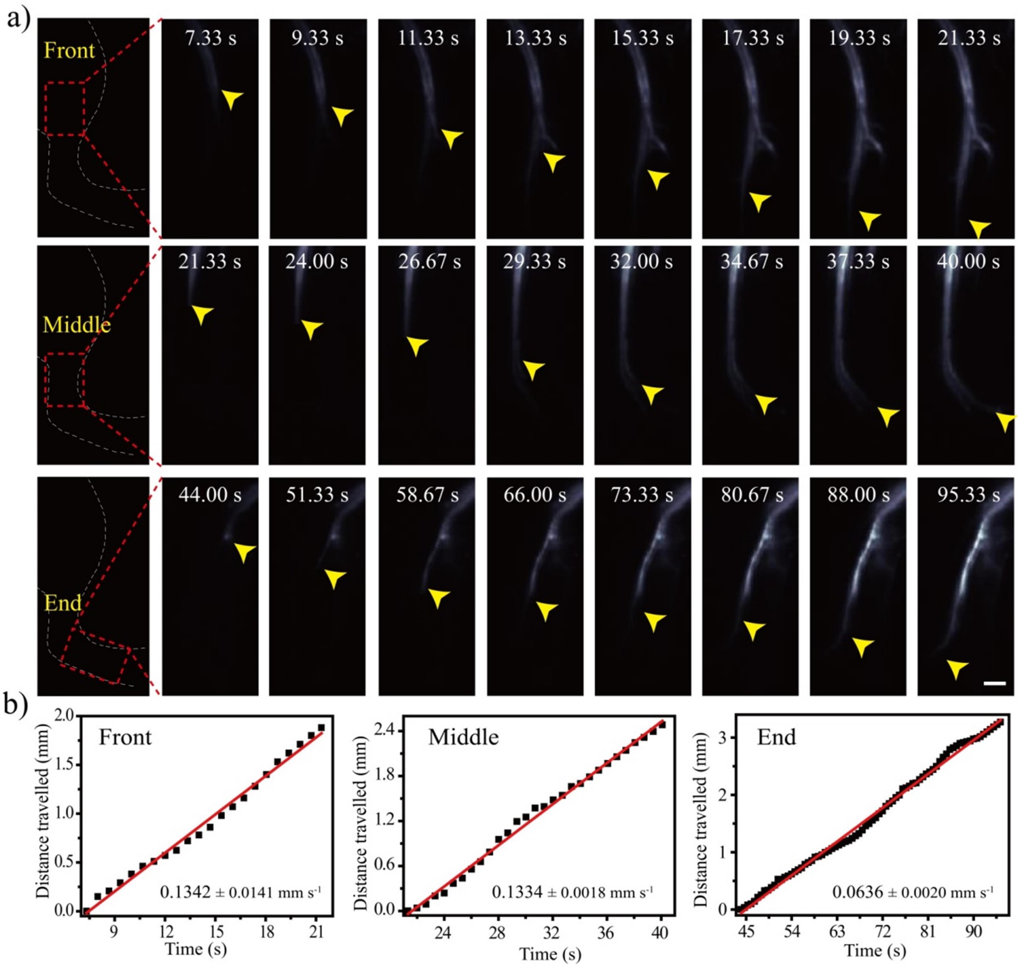

Supplementary Figure 44. Calculation of the ischemic reperfusion blood flow velocity (BFV) in mice hindlimb after clipping for 4 h. **a** NIR-II images of ischemic reperfusion at different time points after clipping for 4 h with LZ-1105 administration (λ_{ex} = 1064 nm, 1400 nm long-pass filter, 300 ms). Yellow arrows indicate the flow front. **b** The corresponding distance travelled by the flow front as a function of time at the front, middle and end site. The slopes of the function were calculated as BFV. When the clipping time was 4 h, the BFV in the front and middle site was comparable, while the BFV in the end site was significantly slower, illustrating the reperfusion in the ischemic hindlimb was blocked slightly. The slope of the function was calculated as blood flow velocity (BFV) and indicats mean ± s.d. derived from n=3 replicated measurements. Scale bar represents 4 mm. Source data underlying b are provided as a Source Data file.

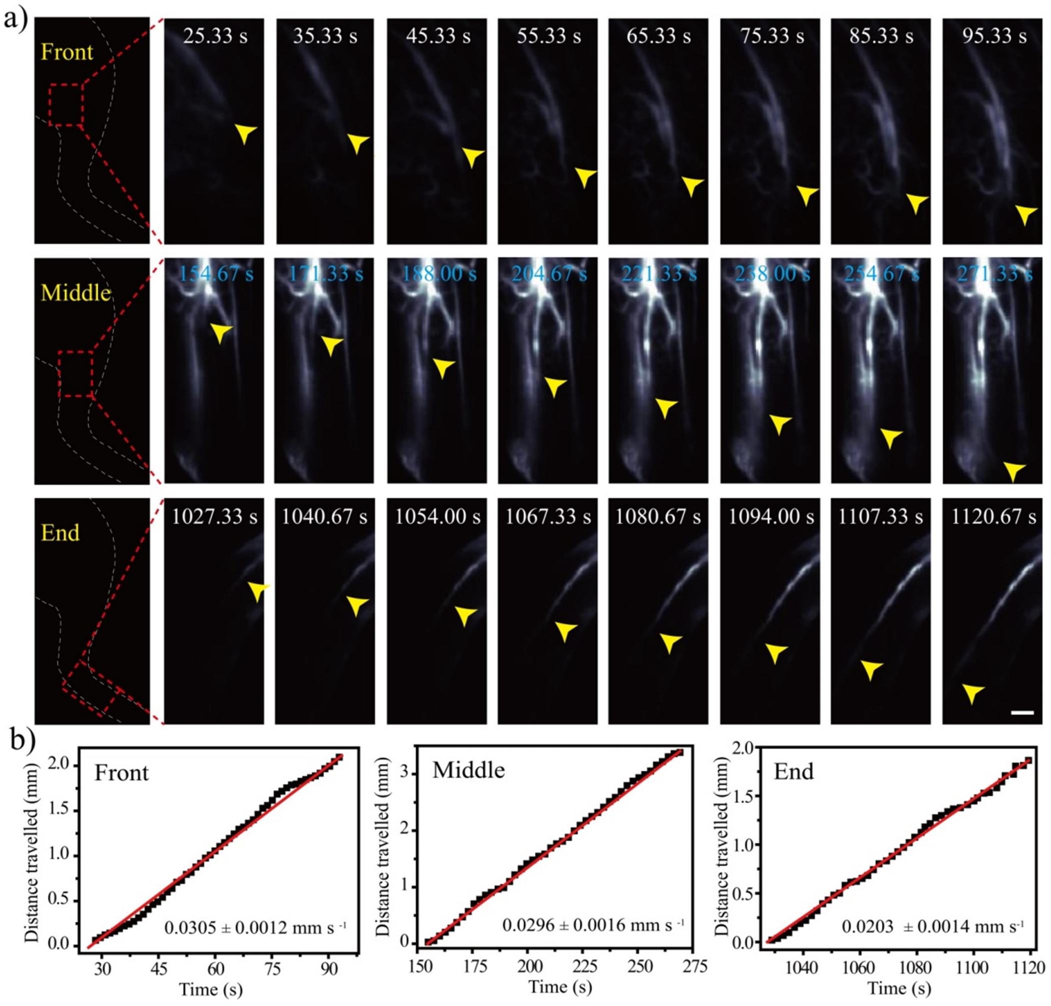

Supplementary Figure 45. Calculation of the ischemic reperfusion blood flow velocity (BFV) in mice hindlimb after clipping for 8 h. **a** NIR-II images of ischemic reperfusion at different time points after clipping for 8 h with LZ-1105 administration (λ_{ex} = 1064 nm, 1400 nm long-pass filter, 300 ms). Yellow arrows indicate the flow front. **b** The corresponding distance travelled by the flow front as a function of time at the front, middle and end site. The slopes of the function were calculated as BFV. When the clipping time was 8 h, the BFV decreased significantly from the front site to the end site, illustrating the reperfusion in the ischemic hindlimb was blocked severely. The slope of the function was calculated as blood flow velocity (BFV) and indicats mean ± s.d. derived from n=3 replicated measurements. Scale bar represents 4 mm. Source data underlying b are provided as a Source Data file.

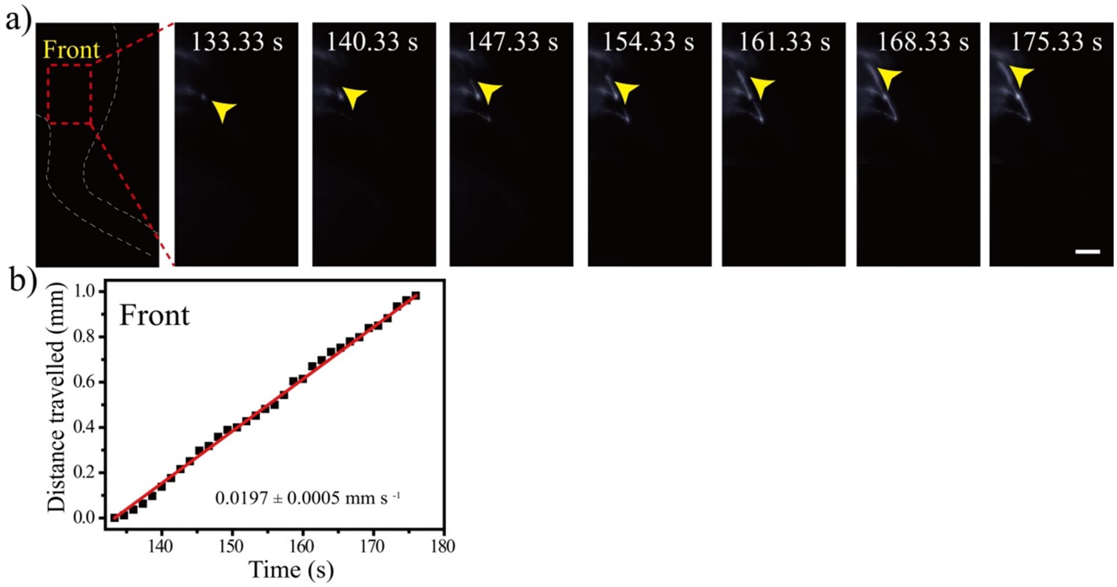

Supplementary Figure 46. Calculation of the ischemic reperfusion blood flow velocity (BFV) in mice hindlimb after clipping for 12 h. **a** NIR-II images of ischemic reperfusion at different time points after clipping for 12 h with LZ-1105 administration (λ_{ex} = 1064 nm, 1400 nm long-pass filter, 300 ms). Yellow arrows indicate the flow front. **b** The corresponding distance travelled by the flow front as a function of time. The slope of the function was calculated as BFV. When the clipping time was 12 h, only the BFV in the front site was detected, while the BFV in the middle and end site were failed to calculate, illustrating the reperfusion in the ischemic hindlimb could not be realized once the clipping time reached 12 h. The slope of the function was calculated as blood flow velocity (BFV) and indicats mean ± s.d. derived from n=3 replicated measurements. Scale bar represents 4 mm. Source data underlying b are provided as a Source Data file.

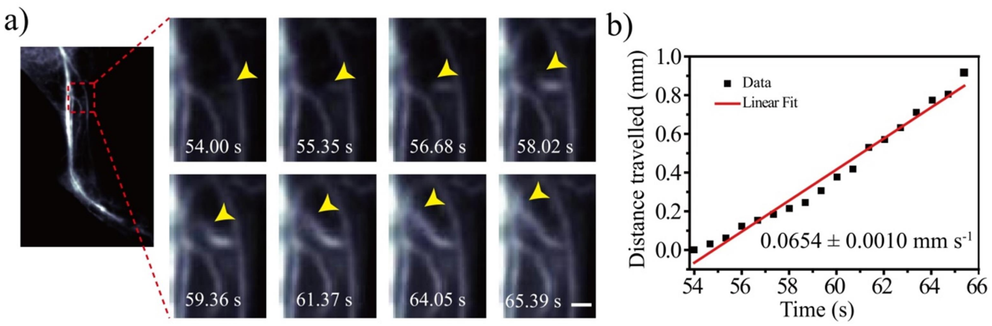

Supplementary Figure 47. Calculation of the ischemic reperfusion blood flow velocity (BFV) of branch vessel in mice hindlimb after clipping for 1 h. **a** NIR-II images shows the flow front (marked by yellow arrows) in the hindlimb of an ischemic mouse. Yellow arrows indicate the flow front. **b** Distance traveled by the flow front as a function of time. The slope of the function was calculated as BFV. The slope of the function was calculated as blood flow velocity (BFV) and indicats mean ± s.d. derived from n=3 replicated measurements. Scale bar represents 0.5 mm. Source data underlying b are provided as a Source Data file.

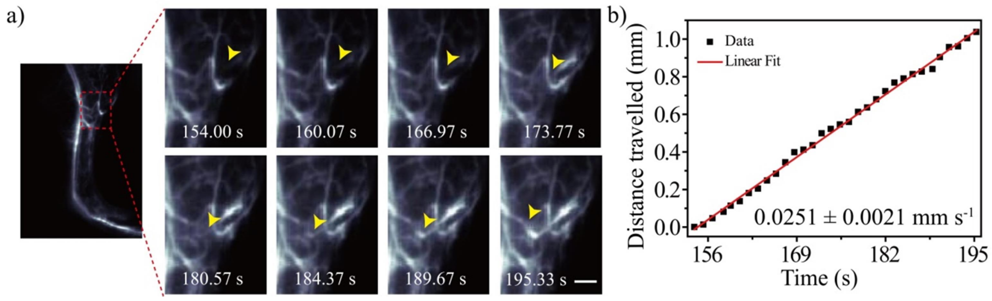

Supplementary Figure 48. Calculation of the ischemic reperfusion blood flow velocity (BFV) of branch vessel in mice hindlimb after clipping for 4 h. **a** NIR-II images shows the flow front (marked by yellow arrows) in the hindlimb of an ischemic mouse. Yellow arrows indicate the flow front. **b** Distance traveled by the flow front as a function of time. The slope of the function was calculated as BFV. The slope of the function was calculated as blood flow velocity (BFV) and indicats mean ± s.d. derived from n=3 replicated measurements. Scale bar represents 0.5 mm. Source data underlying b are provided as a Source Data file.

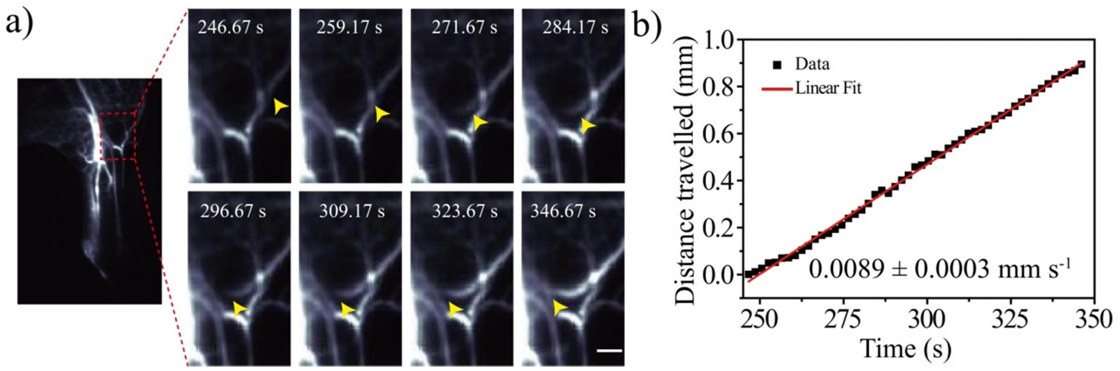

Supplementary Figure 49. Calculation of the ischemic reperfusion blood flow velocity (BFV) of branch vessel in mice hindlimb after clipping for 8 h. **a** NIR-II images shows the flow front (marked by yellow arrows) in the hindlimb of an ischemic mouse. Yellow arrows indicate the flow front. **b** Distance traveled by the flow front as a function of time. The slope of the function was calculated as BFV. The slope of the function was calculated as blood flow velocity (BFV) and indicats mean ± s.d. derived from n=3 replicated measurements. Scale bar represents 0.5 mm. Source data underlying b are provided as a Source Data file.

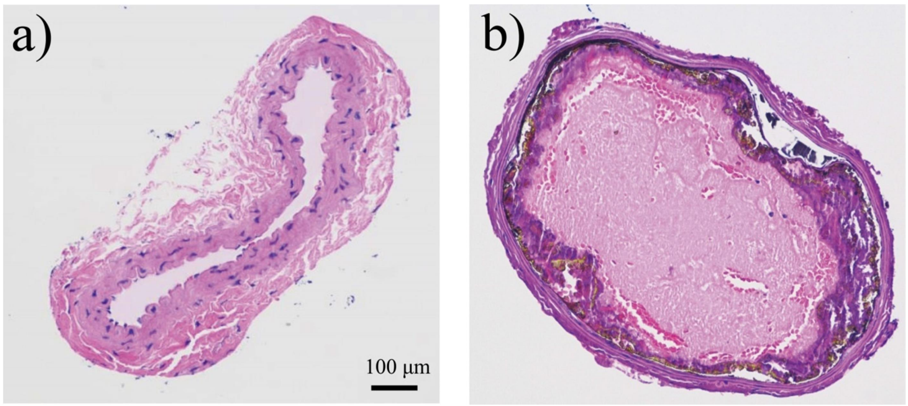

Supplementary Figure 50. Pathology images of carotid artery without (left) and with (right) thrombus. Haematoxylin and Eosin staining of carotid artery without (left) and with (right) the $FeCl_3$ administration. Experimental results prove that successful formation of a thrombus in the right carotid artery. n=3 independent mice experiments.

NMR and MS spectra.

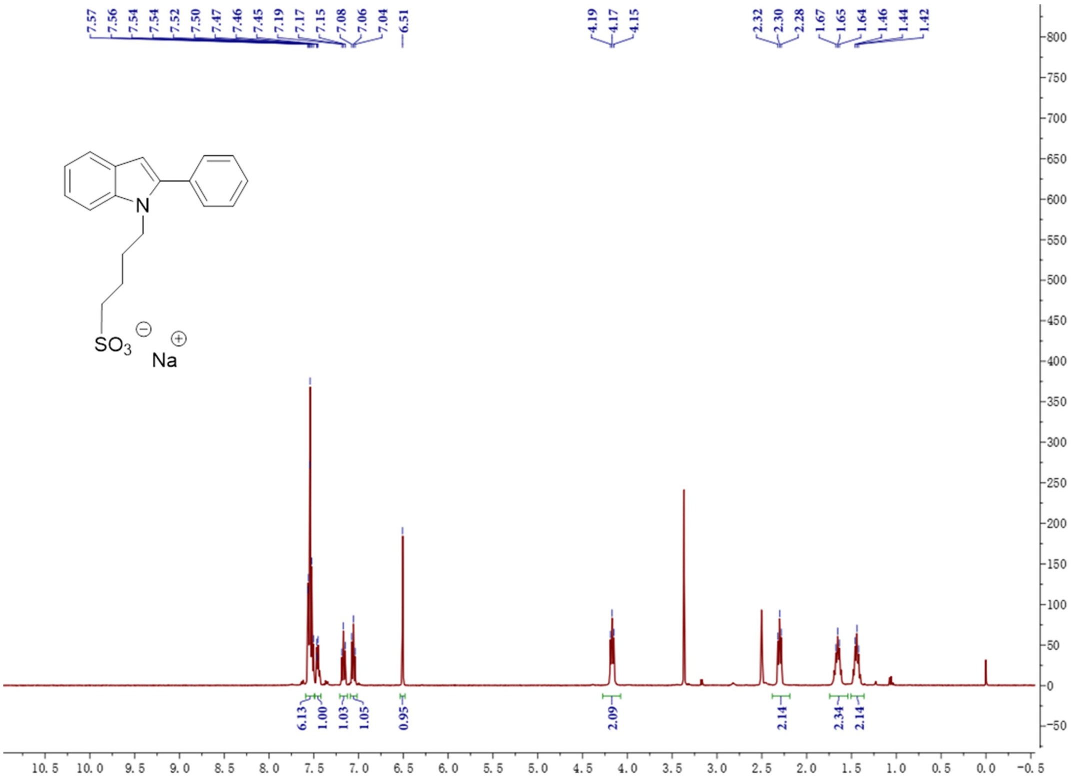

Supplementary Figure 49. The ^{1}H-NMR spectrum of **1** in DMSO-d_6.

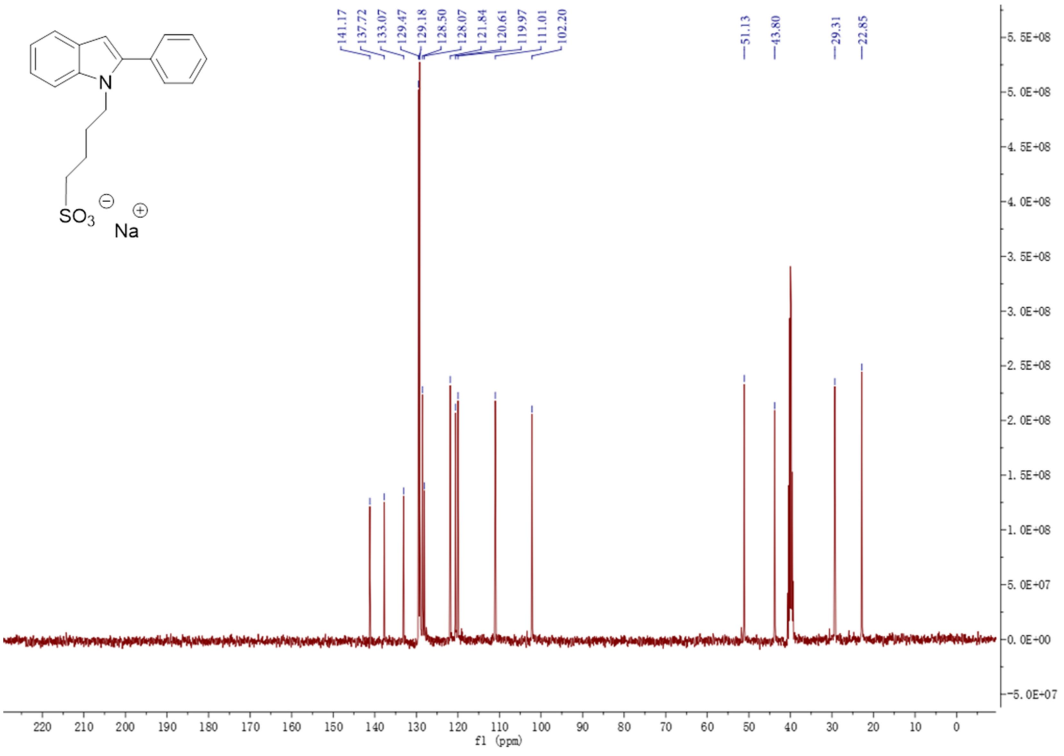

Supplementary Figure 50. The ^{13}C-NMR spectrum of **1** in DMSO-d_6.

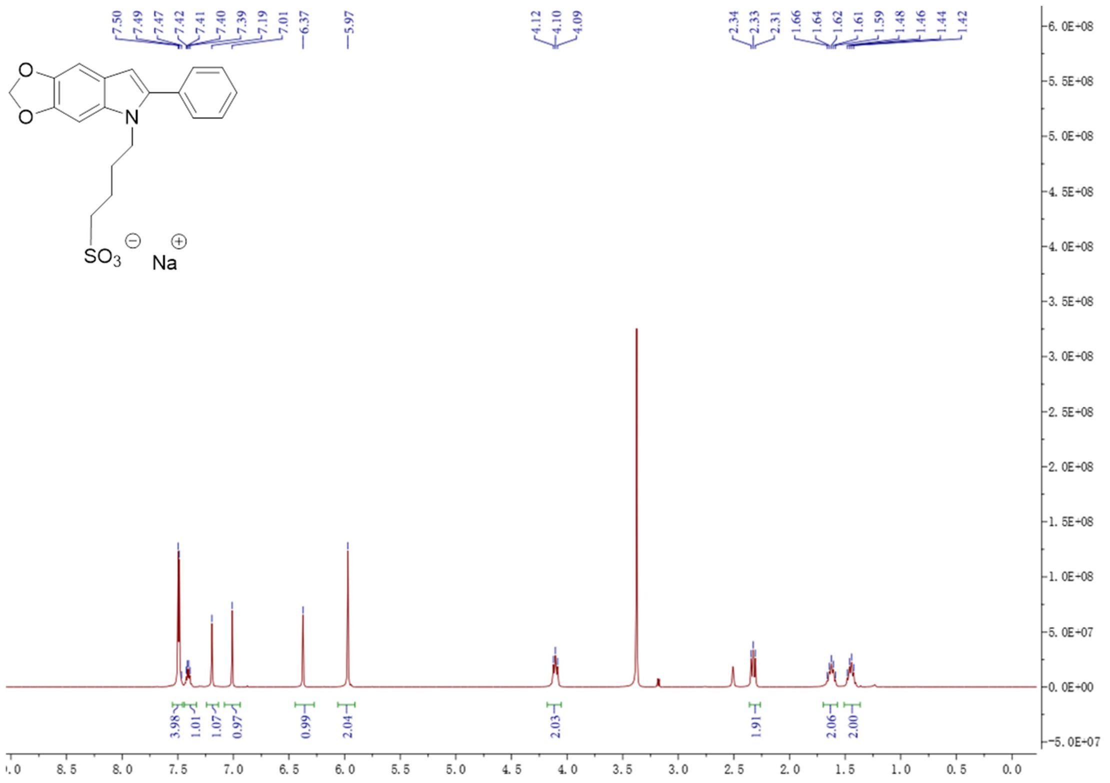

Supplementary Figure 51. The ^{1}H-NMR spectrum of **2** in DMSO-d_6.

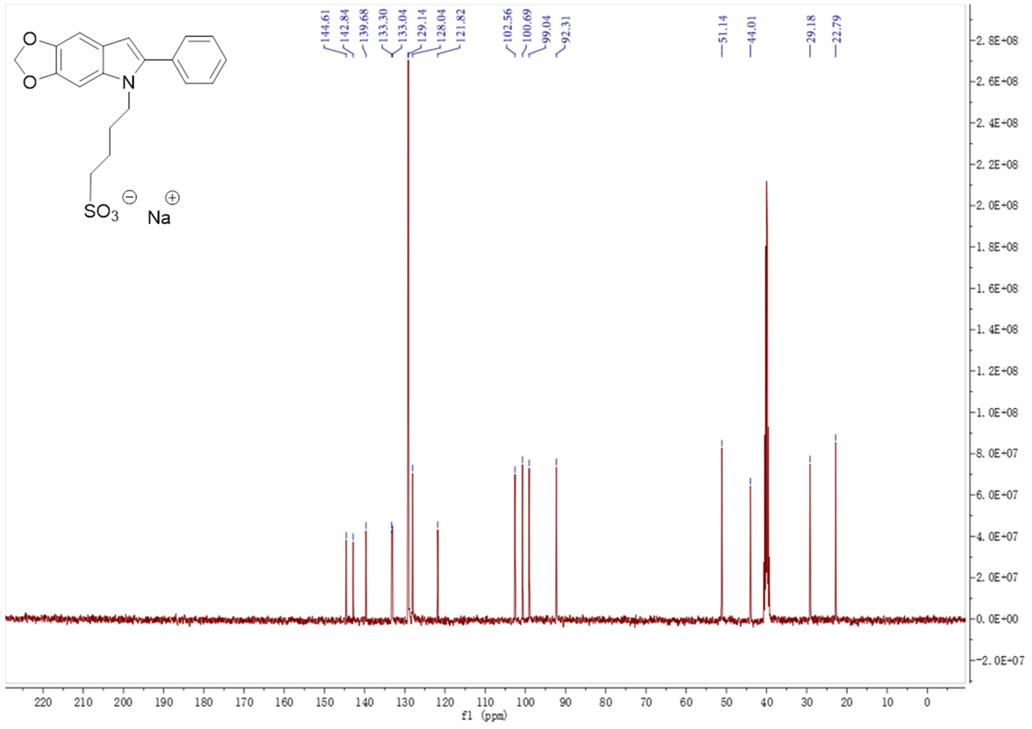

Supplementary Figure 52. The ^{13}C-NMR spectrum of **2** in DMSO-d_6.

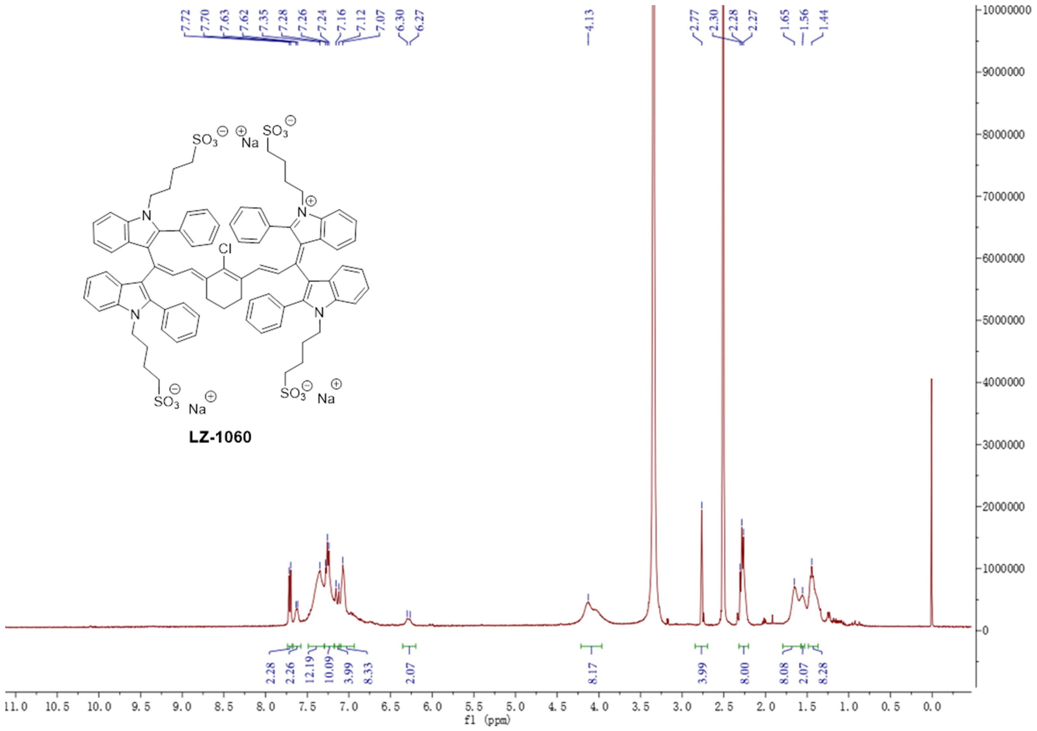

Supplementary Figure 53. The ^{1}H-NMR spectrum of LZ-1060 in DMSO-d_6.

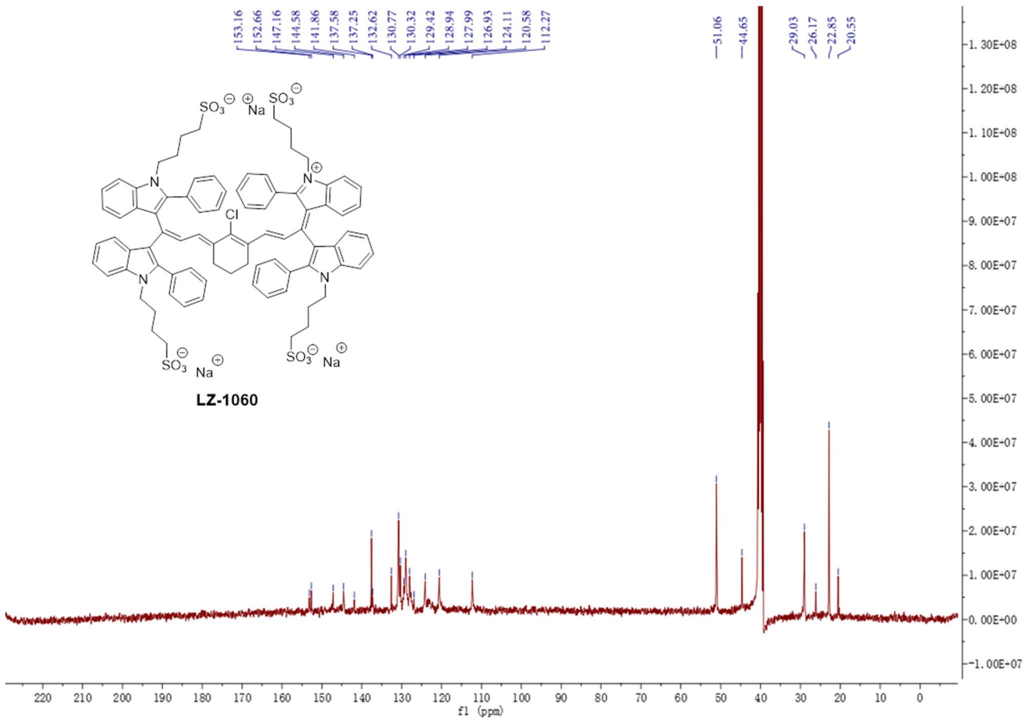

Supplementary Figure 54. The ^{13}C-NMR spectrum of LZ-1060 in DMSO-d_6.

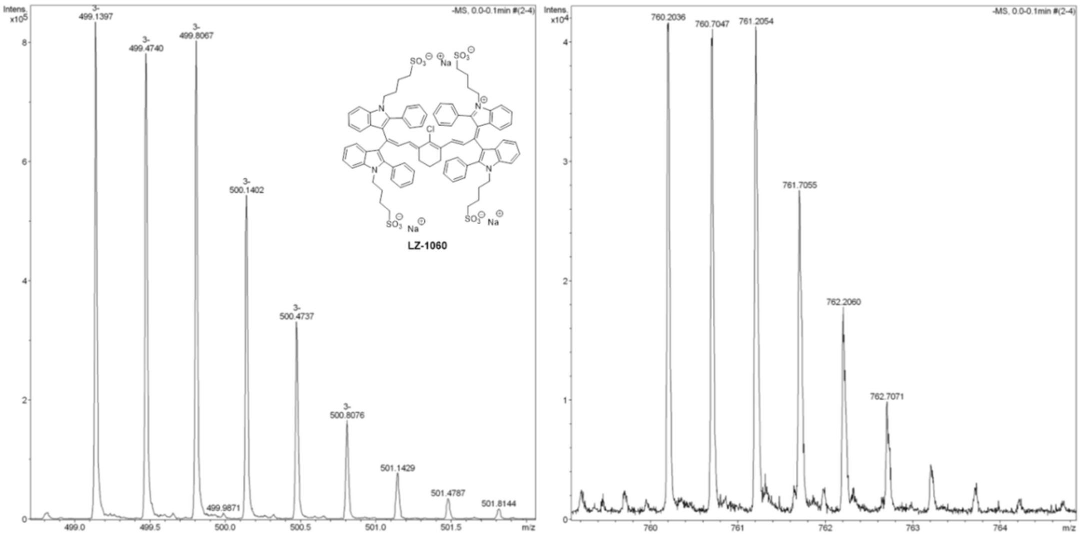

Supplementary Figure 55. The MS of LZ-1060.

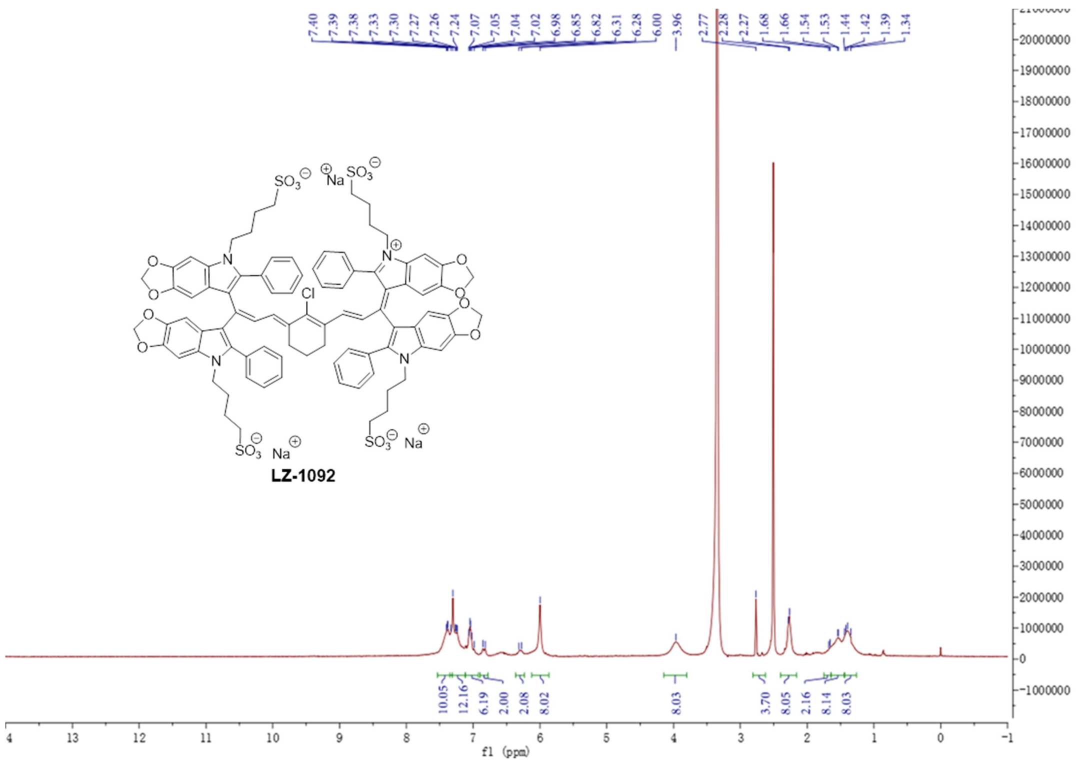

Supplementary Figure 56. The ^{1}H-NMR spectrum of LZ-1092 in DMSO-d_6.

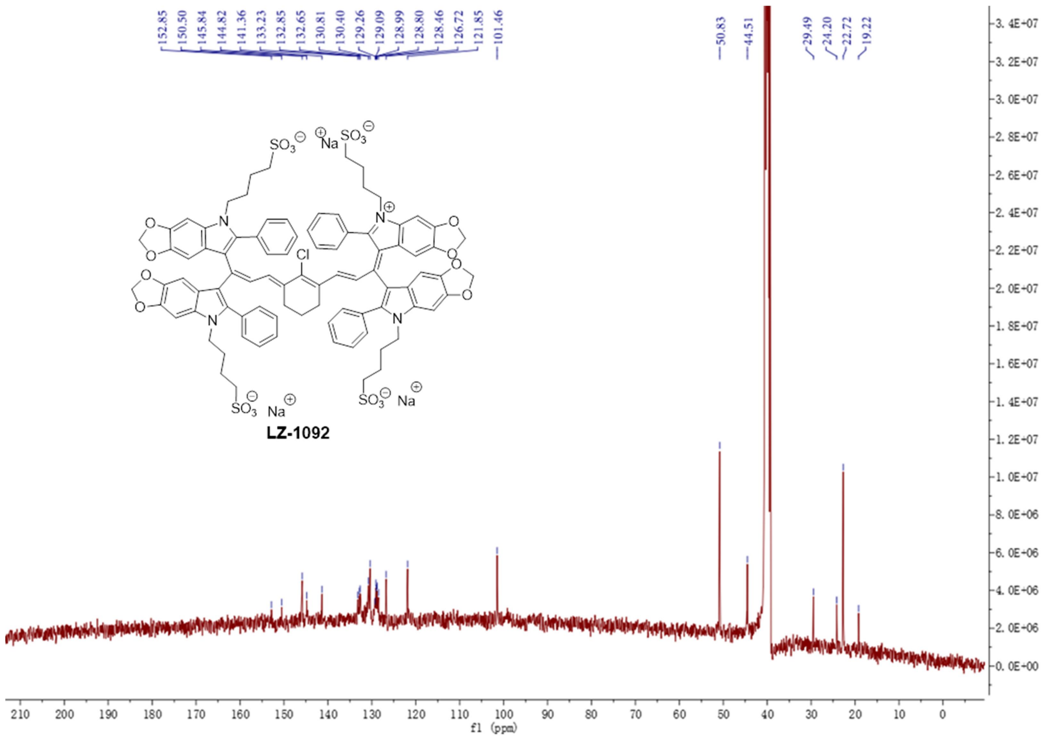

Supplementary Figure 57. The ^{13}C-NMR spectrum of LZ-1092 in DMSO-d_6.

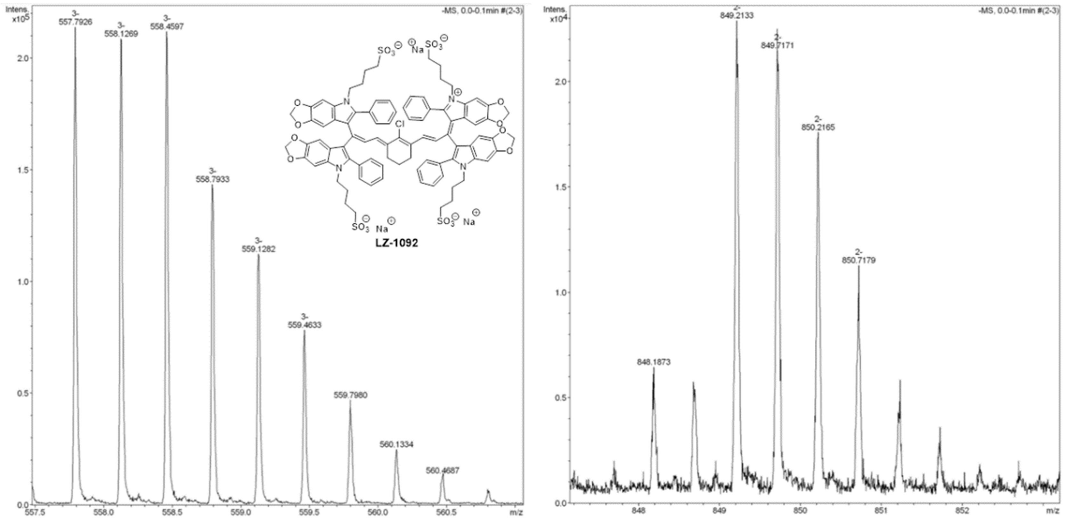

Supplementary Figure 58. The MS of LZ-1092.

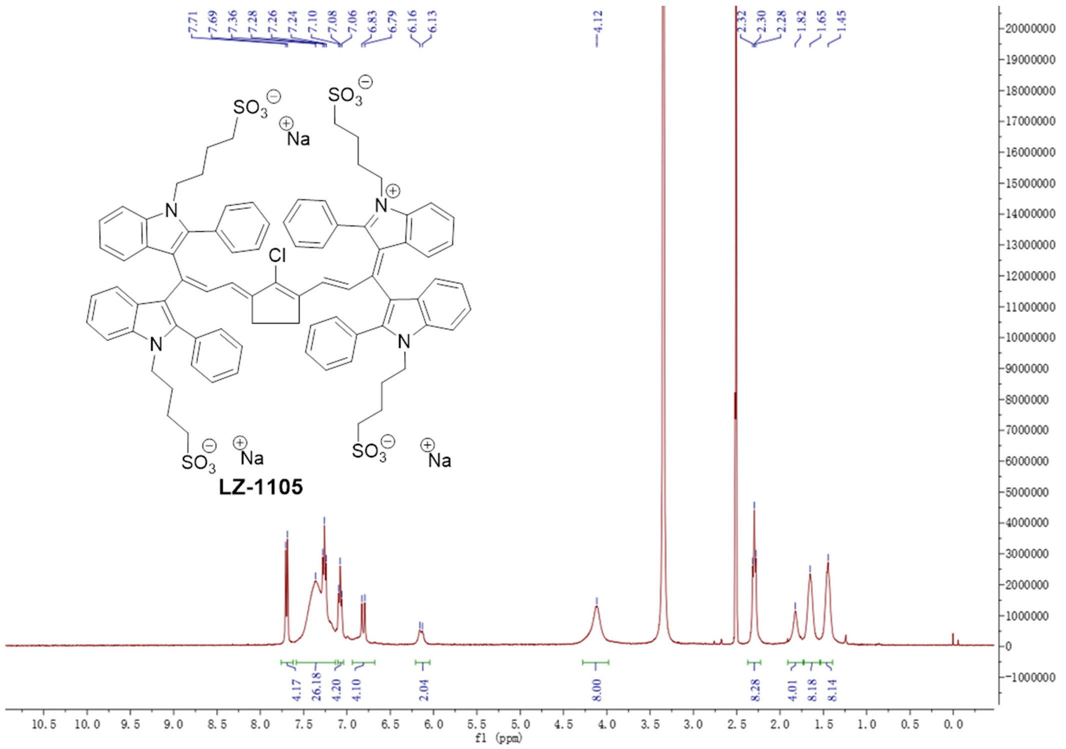

Supplementary Figure 59. The ^{1}H-NMR spectrum of LZ-1105 in DMSO-d_6.

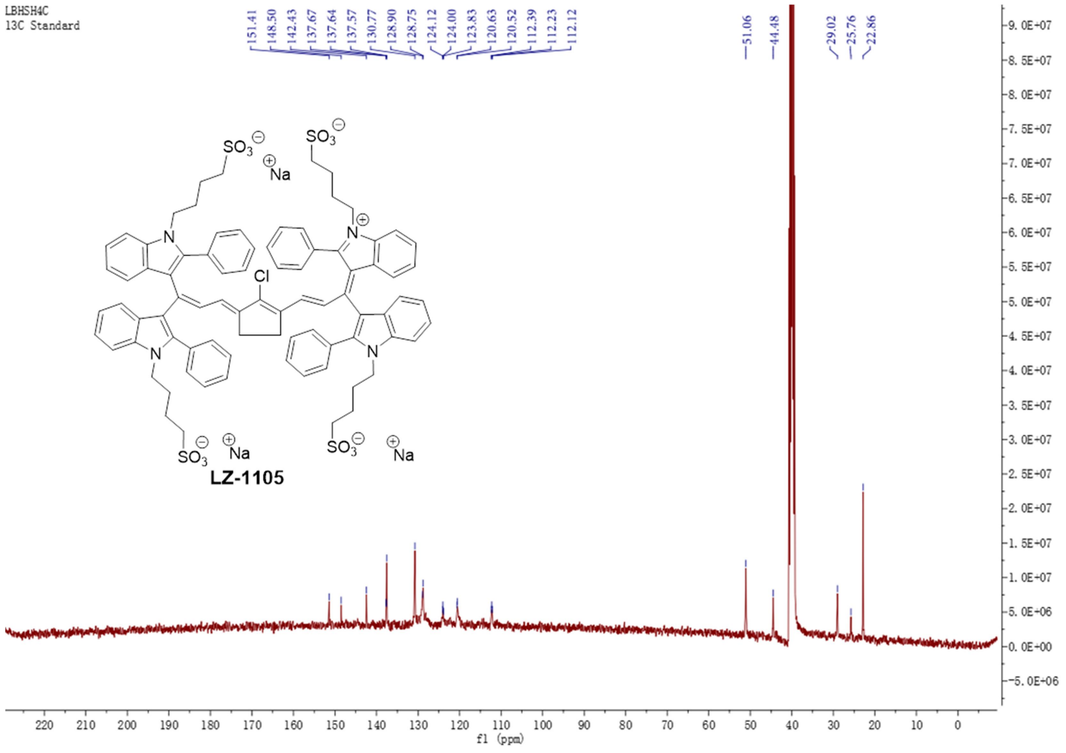

Supplementary Figure 60. The ^{13}C-NMR spectrum of LZ-1105 in DMSO-d_6.

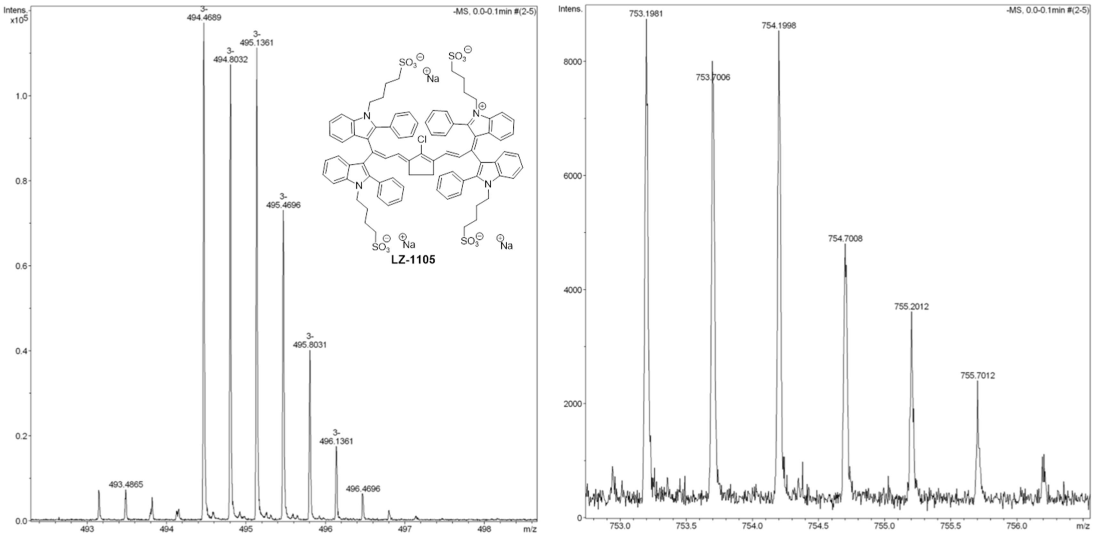

Supplementary Figure 61. The MS of LZ-1105.

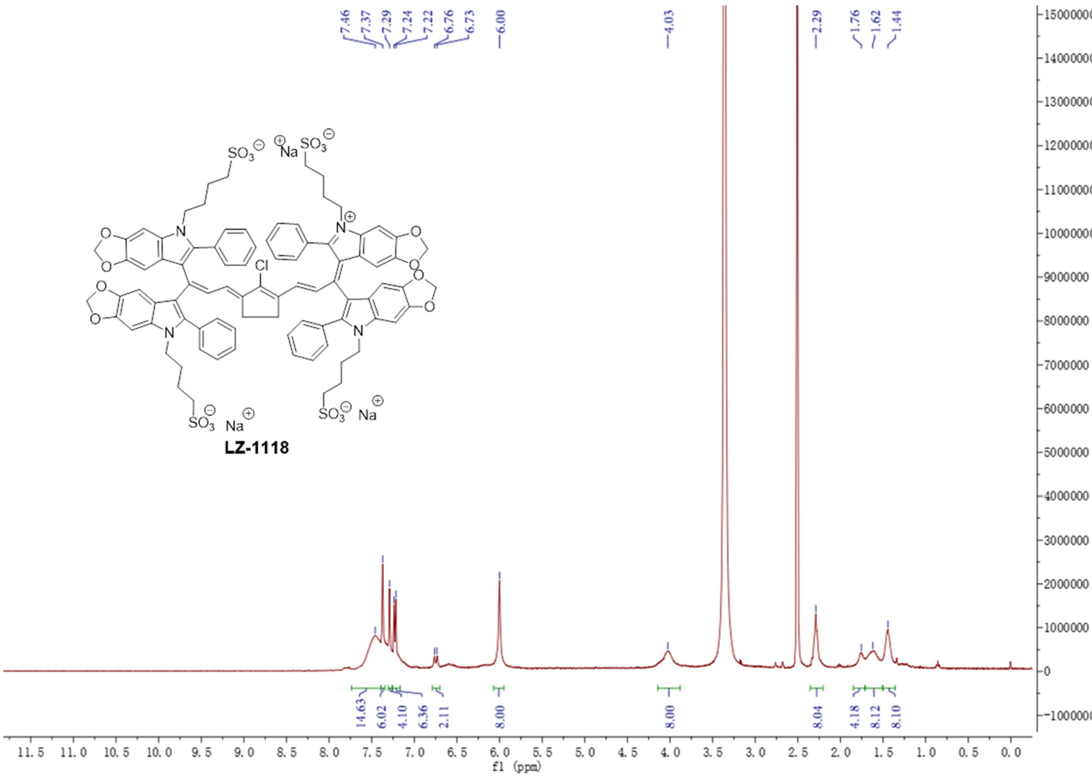

Supplementary Figure 62. The ^{1}H-NMR spectrum of LZ-1118 in DMSO-d_6.

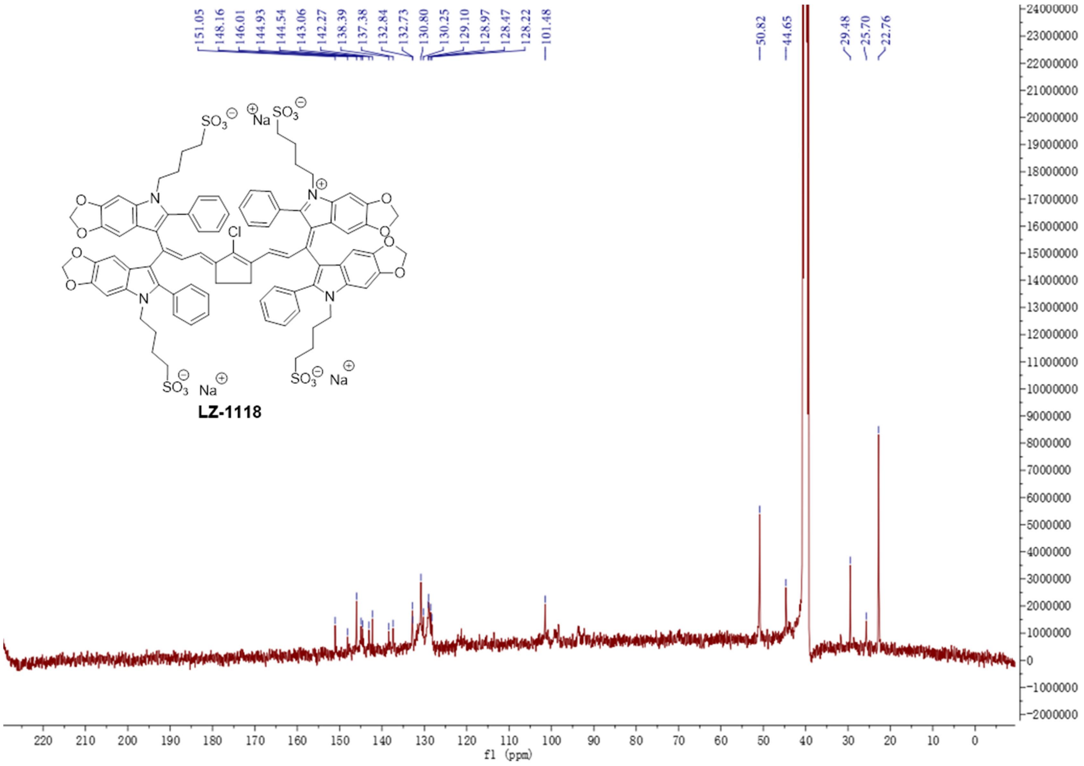

Supplementary Figure 63. The ^{13}C-NMR spectrum of LZ-1118 in DMSO-d_6.

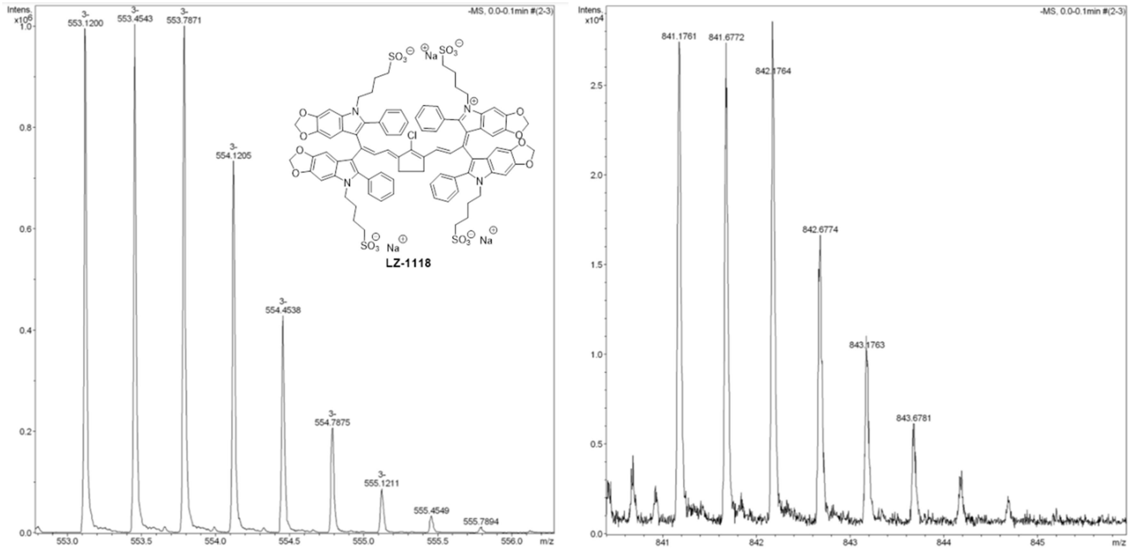

Supplementary Figure 64. The MS of LZ-1118.

Supplementary Tables

Supplementary Table 1. Overview of the NIR-II organic molecular dyes.

NIR-II organic molecular dyes	Blood half-life time (min)	ref
Indocyanine green (ICG)	5	2
IR-12N3	12	3
CH1055-PEG	60	4
LZ-1105	192	This work

Supplementary Table 2. Optical and Photochemical Properties of LZ dyes

Dye	Solvent	λ_{abs} (nm)[a]	λ_{em} (nm)[b]	$\varepsilon_{max} \times 10^5$ (cm^{-1} mol • L^{-1})	Φ_f (%)[c]
LZ-1060	CH_3OH	1003	1038	1.47	1.53
	CH_3CH_2OH	1009	1033	1.60	1.94
	DMSO	1023	1059	1.28	3.55
	PBS	1021	1060	1.08	0.06
LZ-1092	CH_3OH	1013	1050	1.75	0.80
	CH_3CH_2OH	1017	1043	1.62	0.97
	DMSO	1029	1065	1.21	1.28
	PBS	1035	1092	1.06	0.02
LZ-1105	CH_3OH	1034	1075	1.66	1.22
	CH_3CH_2OH	1041	1069	1.57	2.66
	DMSO	1058	1100	1.48	3.89
	PBS	1041	1105	1.01	0.03
LZ-1118	CH_3OH	1045	1085	1.81	0.56
	CH_3CH_2OH	1051	1084	1.99	0.53
	DMSO	1069	1105	1.46	0.72
	PBS	1052	1118	1.08	0.01

[a] The maximal absorption wavelength of the dye. [b] The maximal emission wavelength of the dye. [c] The relative fluorescence quantum yield (0.05%) by using IR-26 in 1,2-dichloroethane as a reference system.

Supplementary Table 3. The characterization of the typical cyanine dyes in PBS (pH =7.4).

Dye	λ_{abs} (nm) [a]	$\varepsilon_{max} \times 10^5$ (cm^{-1} mol • L^{-1})	Ref.
ICG	780	1.5	2
IRDye 800CW	776	2.4	3
Cy7-Cl	783	2.0	5

[a] The maximal absorption wavelength of the dye.

Supplementary Table 4. The binging parameters (L mol^{-1})($\times 10^5$) of LZ-1105 and ICG with BSA and bovine fibrinogen.

	BSA	Bovine fibrinogen
ICG	1.27 ± 0.07	N/A
LZ-1105	N/A	0.18 ± 0.02

“N/A” means not measureable by ITC.

Supplementary Table 5. The characterization of the recovery starting time point and blood flow velocity (BFV) of vessel marked at the front site with yellow circles on ischemic hindlimb after different clipping times.

Clip vessel time (h)	Recovery starting time of front site (s)	BFV (mm s^{-1})
1	4.67	0.8269 ± 0.0221
4	7.33	0.1342 ± 0.0141
8	25.33	0.0305 ± 0.0012
12	133.33	0.0197 ± 0.0005

Supplementary Table 6. The characterization of the recovery starting time point and blood flow velocity (BFV) of vessel marked at the middle site with yellow circles on ischemic hindlimb after different clipping times.

Clip vessel time (h)	Recovery starting time of middle site (s)	BFV (mm s^{-1})
1	10.67	0.8269 ± 0.0221
4	21.33	0.1334 ± 0.0018
8	154.67	0.0296 ± 0.0016
12	N/A	N/A

"N/A" means not measureable by NIR-II imaging.

Supplementary Table 7. The characterization of the recovery starting time point and blood flow velocity (BFV) of vessel marked at the end site with yellow circles on ischemic hindlimb after different clipping times.

Clip vessel time (h)	Recovery starting time of end site (s)	BFV (mm s^{-1})
1	14.67	0.8269 ± 0.0221
4	44.00	0.0636 ± 0.0020
8	1027.33	0.0203 ± 0.0014
12	N/A	N/A

"N/A" means not measureable by NIR-II imaging.

Supplementary Table 8. The characterization of the recovery starting time point and blood flow velocity (BFV) of branch vessel on ischemic hindlimb after different clipping times.

Clip vessel time (h)	Recovery starting time (s)	BFV (mm s^{-1})
1	54.00	0.0654 ± 0.0010
4	154.00	0.0251 ± 0.0021
8	246.67	0.0089 ± 0.0003
12	N/A	N/A

"N/A" means not measureable by NIR-II imaging.

Supplementary Methods

Materials:

2-Phenylindole (98 %), 5,6-methylenedioxy-2-phenylindole (97 %), sodium hydride (60 %), anhydrous tetrahydrofuran (99 %), butanesultone (99 %), were purchased from Adamas. Sodium hydroxide (NaOH, 96 %), ethanol, methanol, dimethylsulfoxide were obtained from Beijing Chemical Reagents Co. Ltd. Isopropyl alcohol (99 %), acetyl chloride (99 %), acetic anhydride (99 %), ether (99 %), acetonitrile (99 %), bull serum albumin (BSA) and bovine fibrinogen were purchased from Aladdin Industrial Inc. Trypsin-EDTA, Penicillin-Streptomycin Solution (100X) and SDS-PAGE gel preparation kit (7.5%) were purchased from MesGen Biotech. Recombinant tissue plasminogen activator (rt-PA) was purchased from Boehringer Ingelheim Pharma GmbH & Co. Plasmin (≥ 2.0 units mg^{-1} protein) was purchased from Sigma-Aldrich. The phosphate buffered solutions (PBS, pH = 7.4) was purchased from Gelest Inc. Silica gel plates and column chromatography were purchased from the Qingdao Ocean Chemicals. All chemicals were used as received without any further purification.

The NMR spectra were measured with a Bruker AV 400 NMR spectrometer, using TMS as an internal standard. The high-resolution mass spectra (HRMS) were measured with in ESI mode using Bruker maxis UHR-TOF mass spectrometer. Isothermal titration calorimetry (ITC) experiments were conducted on a MicroCal VP-ITC system at 20.00 ± 0.01°C. Absorption spectra were collected by using a PerkinElmer Lambda 750S UV-visible-NIR spectrometer with a 2000 nm min^{-1} scan rate. The emission spectra were obtained on an Edinburgh Instruments F-980 fluorescence spectrometer or home-built liquid-nitrogen-cooled InGaAs array detector (Princeton Instruments, NIRvana 640LN). Quartz cuvettes (1 cm) were used for absorbance and emission

measurements. Absorption coefficients were calculated with serial dilutions in different solvents. Ultrasound measurements were performed using the ACUSON S3000 ultrasound system (Siemens, Erlangen, Germany) equipped with a linear probe (18 MHz). Reversed-phase high performance liquid chromatographic (RP-HPLC) analyses were performed on an Waters equipped with a UV detector and an Atlantls T3 OBD-C18 RP (10 × 250 mm) column, with methanol and H2O (0.1% of trifluoroacetic acid) as the eluent.

General Procedure for synthesis of compounds **1** and **2**: 2-Phenylindole (2.0 mmol, 386 mg) or 5,6-Methylenedioxy-2-phenylindole (2.0 mmol, 415 mg) and 60% Sodium hydride (2.1 mmol, 84 mg) were mixed in anhydrous tetrahydrofuran (10 mL) and stirred at 0 °C for 20 min. To this solution was added butanesultone (2.0 mmol, 272 mg) and then heated at 100 °C for 2 h. After cooling, the solution was treated with isopropyl alcohol. The white solid was collected by filtration to afford compounds **1** or **2** (yield 95-99%).

Compound **1:** ^{1}H NMR (400 MHz, DMSO-d_6) δ 7.41-7.36 (m, 2H), 7.05-7.01 (m, 1H), 6.96-6.92 (m, 1H), 6.17 (s, 1H), 4.10-4.07 (t, 2H), 2.49-2.46 (m, 2H), 2.40 (s, 3H), 1.73-1.68 (m, 2H), 1.65-1.59 (m, 2H).^{13}C NMR (101 MHz, DMSO-d_6) δ 136.9, 136.8, 128.0, 120.4, 119.5, 119.2, 109.8, 99.8, 51.4, 42.8, 29.5, 23.1, 12.9.

Compound **2:** ^{1}H NMR (400 MHz, DMSO-d_6) δ 7.57-7.50 (m, 6H), 7.47-7.45 (m, 1H), 7.19-7.15 (m, 1H), 7.08-7.04 (m, 1H), 6.51 (s, 1H), 4.19-4.15 (t, 2H), 2.32-2.28 (m, 2H), 1.67-1.64 (m, 2H), 1.46-1.42 (m, 2H).^{13}C NMR (101 MHz, DMSO-d_6) δ 141.2, 137.7, 133.1, 129.5, 129.2, 128.5, 128.1, 121.8, 120.6, 119.9, 111.0, 102.2, 51.1, 43.8, 29.3, 22.9.

General Procedure for synthesis of dyes LZ-1060, LZ-1092, LZ-1105, LZ-1118: Compound **1** (4.3

mmol, 1.14 g) and acetyl chloride (2.2 mmol, 172 mg) in acetic anhydride (20 mL) was heated at 55°C for 4h. N-[(3-(Anilinomethylene)-2-chloro-1-cyclohexen-1-yl) methylene] aniline monohydrochloride (compound **4,** 2.2 mmol, 720 mg) was added to mixed solution, and then heated at 100 °C for 1.5 h. After cooling, the solution was treated with ether (60 mL). The precipitate was collected by filtration. The product was purified by preparative RP HPLC (Waters C18 (5 µm, 250 mm × 20 mm) (yield 90-93%).

Compound LZ-1060**:** ^{1}H NMR (400 MHz, DMSO-d_6) δ 7.72-7.70 (d, 2H), 7.63-7.62 (d, 2H), 7.35 (s, 12H), 7.28-7.24 (m, 10H), 7.16-7.12 (m, 4H), 7.09-7.04 (m, 8H), 6.30-6.27 (d, 2H), 4.13 (m, 8H), 2.77 (m, 4H), 2.30-2.27 (t, 8H), 1.65 (s, 8H), 1.56 (s, 2H), 1.48-1.41 (m, 8H). ^{13}C NMR (101 MHz, DMSO-d_6) δ 153.2, 152.7, 147.2, 144.6, 141.9, 137.6, 137.3, 132.6, 130.8, 130.3, 129.4, 128.9, 127.9, 126.9, 124.1, 120.6, 112.3, 51.1, 44.7, 29.0, 26.2, 22.9, 20.6. HRMS (ESI) $[C_{84}H_{78}ClN_4O_{12}S_4Na]^{2-}$: calculated: 760.2048, measured: 760.2036, $[C_{84}H_{78}ClN_4O_{12}S_4]^{3-}$: calculated: 499.1401, measured: 499.1397.

Compound LZ-1092: ^{1}H NMR (400 MHz, DMSO-d_6) δ 7.40-7.38 (m, 10H), 7.33-7.24 (m, 12H), 7.07-6.98 (m, 6H), 6.85-6.82 (d, 2H), 6.31-6.28 (d, 2H), 6.00 (s, 8H), 3.96 (s, 8H), 2.77 (m, 4H), 2.28-2.27 (m, 8H), 1.68-1.66 (m, 2H), 1.54-1.53 (m, 8H), 1.44-1.34 (m, 8H). ^{13}C NMR (101 MHz, DMSO-d_6) δ 152.9, 150.5, 145.8, 144.8, 141.4, 133.2, 132.9, 132.6, 130.8, 130.4, 129.3, 129.1, 128.9, 128.8, 128.4, 126.7, 121.8, 101.5, 50.8, 44.5, 29.5, 29.2, 28.9, 24.2, 22.7, 19.2. HRMS (ESI) $[C_{88}H_{78}ClN_4O_{20}S_4Na]^{2-}$: calculated: 848.1874, measured: 848.1873, $[C_{88}H_{78}ClN_4O_{20}S_4]^{3-}$: calculated: 557.7926, measured: 557.7932.

Compound LZ-1105: ^{1}H NMR (400 MHz, DMSO-d_6) δ 7.71-7.69 (d, 4H), 7.36-7.24 (m, 26H), 7.10-7.06 (d, 4H), 6.83-6.79 (d, 4H), 6.15-6.13 (d, 2H), 4.12 (m, 8H), 2.32-2.28 (m, 8H), 1.82 (s, 4H),

1.67-1.64 (m, 8H), 1.46-1.45 (m, 8H). ^{13}C NMR (101 MHz, DMSO-d_6) δ 151.4, 148.5, 142.4, 137.7, 137.6, 137.6, 130.8, 128.9, 128.7, 124.1, 124.0, 123.8, 120.6, 120.5, 112.4, 112.2, 112.1, 51.1, 44.5, 29.0, 25.8, 22.9. HRMS (ESI) $[C_{83}H_{76}N_4O_{12}S_4Na]^{2-}$: calculated: 753.1970, measured: 753.1981, $[C_{83}H_{76}N_4O_{12}S_4]^{3-}$: calculated: 494.4683, measured: 494.4689.

Compound LZ-1118: ^{1}H NMR (400 MHz, DMSO-d_6) δ 7.49-7.46 (m, 14H), 7.37-7.35 (m, 6H), 7.29-7.27 (m, 4H), 7.24-7.22 (m, 6H), 6.76-6.73 (d, 2H), 6.00 (s, 8H), 4.03 (s, 8H), 2.31-2.27 (t, 8H), 1.77-1.76 (m, 4H), 1.62-1.60 (m, 8H), 1.45-1.42 (m, 8H). ^{13}C NMR (101 MHz, DMSO-d_6) δ 151.1, 148.2, 146.0, 144.9, 144.5, 143.1, 142.3, 138.4, 137.4, 132.8, 132.7, 130.8, 130.2, 129.1, 128.9, 128.5, 128.2, 101.5, 50.8, 44.7, 29.5, 25.7, 22.8. HRMS (ESI) $[C_{87}H_{76}ClN_4O_{20}S_4Na]^{2-}$: calculated: 841.1766, measured: 841.1761, $[C_{87}H_{76}ClN_4O_{20}S_4]^{3-}$: calculated: 553.1214, measured: 553.1200.

Supplementary Notes

Supplementary Note 1

Optimizing the signal collection for ICG and LZ-1105 NIR imaging

Various long-pass (850-, 1000-, 1100-, 1200-, 1300- and 1400 nm) filters was used for in-vitro and in-vivo ICG imaging.

First, single ICG-filled (mixed with blood, [ICG] = 10 μM) capillary immersed into 1% Intralipid was excited at 808 nm and the emission was collected by different long-pass filters at varied Intralipid depths (Supplementary Figure 11-12). The imaging signal intensity with different long-pass filters at 0 mm penetration depth was almost the same by adjusting the laser's working power density. With increase of penetration depth, attenuation of signal intensity and blurring of capillary profiles were observed for all images, and only images acquired beyond 1300 nm

resolved sharp edges of the capillary at a depth up to 5.5 mm. 1300 nm long-pass filter group (19.0) exhibited a 10.6-fold signal-to background ratio (SBR) than that of 850 nm long-pass filter group (1.8) under 1.0 mm depth. Besides, FWHM, which was hired to evaluate the resolving ability, was observed to increase more obviously by using 850 nm long-pass filter. These results illustrated that ICG imaging acquired beyond 1300 nm exhibited higher signal-to-background ratio, resolution and deeper penetration compared to that of 850 nm in the presence of biotissue.

To further investigate the resolving ability in the presence of biotissue with 1300 nm long-pass filter, two ICG-filled (mixed with blood, [ICG] = 10 μM) capillaries were chosen to mimic adjacent blood vessels in vivo. Two capillaries immersed into 1% Intralipid were excited at 808 nm and the emission was collected by different long-pass filters at varied depths (Supplementary Figure 13). Similar with the single capillary imaging, attenuation of image intensities and blurring of capillary profiles were observed for all images with increase of penetration depth. Contrast of images acquired by various long-pass filters under different depth was calculated. Contrast of various long-pass filters groups was comparable when penetration depth was 0 mm. However, contrast of 850 nm long-pass group dropped below 0.06 under 3.0 mm depth, and the two capillaries could not be resolved from each other. In contrary, contrast of 1300 nm long-pass group stay above 0.2 even under 6.5 mm depth, demonstrating that images acquired by 1300 nm long-pass filter exhibited higher resolution in the presence of biotissue. Next, mice skin was used to repeat the above experiment and similar results could be obtained that ICG bioimaging has higher SBR and contrast by using longer wavelength long-pass filter (Supplementary Figure 14).

Encouraged by the above results, we further investigated the SBR, resolution and contrast of ICG

for in vivo imaging with various long-pass filters. Different 808 nm laser power density was used during the imaging to obtain the same intensity at same signal ROI. As a result, background noise and FWHM decreased while SBR and contrast increased when using the longer long-pass filter, illustrating the superior bioimaging results could be achieved by long-pass filter. When 1400 nm long-pass filter was used, SBR, contrast and FWHM reached the optimum (SBR: 2.5, contrast: 0.29, FWHM: 132 μm) (Supplementary Figure 15-16). However, the laser power density of 808 nm reached 500 mW cm^{-2}, which could lead to strong heating effect of the tissue (Supplementary Figure 17). Same results were obtained in the bioimaging of hindlimb (Supplementary Figure 18-19). Thus 1300 nm long-pass filter was chosen as the proper long-pass filter for ICG imaging. Furthermore, there is a similar work which reported that higher SBR and contrast in NIR-II imaging was obtained with longer filters after the administration of ICG[2,6].

In addition, the proper (1400 nm) long-pass filter for LZ-1105 was also selected by the above method. The data were shown as Supplementary Figure 20-23. Meanwhile, the 1064 nm NIR-II excitation has negligible thermal effect on the tissue under the power density (70 mW cm^{-2}) used for in vivo bioimaging (Supplementary Figure 17). Thus, the proper long-pass filters were used for both ICG (1300 nm long-pass filter) and LZ-1105 (1400 nm long-pass filter) during the imaging. The comparison between ICG and LZ-1105 is fair, and LZ-1105 imaging is superior to ICG for in-vivo bioimaging.

Supplementary References

1. Zauner, G. et al. Glycoproteomic Analysis of Human Fibrinogen Reveals Novel Regions of O-Glycosylation. *J. Proteome Res.* **11**, 5804-5814 (2012).
2. Carr, J.A., *et al.* Shortwave infrared fluorescence imaging with the clinically approved near-infrared dye indocyanine green. *Proc. Natl. Acad. Sci. USA* **115**, 4465-4470 (2018).
3. Zhu, S. *et al.* Repurposing Cyanine NIR-I Dyes Accelerates Clinical Translation of Near-Infrared-II (NIR-II) Bioimaging. *Adv. Mater.* **30**, 1802546 (2018).
4. Antaris, A.L. *et al.* A small-molecule dye for NIR-II imaging. *Nat. Mater.* **15**, 235-242 (2016).
5. Li, B.H. *et al.* A novel sensor for the detection of alkaline phosphatase activity based on the self-assembly of Eu^{3+}-doped oxide nanoparticles and heptamethine cyanine dye. *Sens. Actuators, B* **233**, 479-485 (2016).
6. Starosolski, Z. *et al.* Indocyanine green fluorescence in second near-infrared (NIR-II) window. *PLOS ONE* **12**, e0187563 (2017).
