# Supplementary material for: Organic NIR-II molecule with long blood half-life for in vivo dynamic vascular imaging
Source: Nat Commun. 2020 Jun 18;11:3102. doi: 10.1038/s41467-020-16924-z (PMC7303218; doi:10.1038/s41467-020-16924-z)
Supplement: Supplementary file 3 — Description of Additional Supplementary Files [file 41467_2020_16924_MOESM3_ESM.docx]

File Name: Supplementary Movie 1

Description: Ischemic reperfusion in hindlimb after clipping for 1 h with LZ-1105 administration.

File Name: Supplementary Movie.2

Description: Ischemic reperfusion in hindlimb after clipping for 4 h with LZ-1105 administration.

File Name: Supplementary Movie.3

Description: Ischemic reperfusion in hindlimb after clipping for 8 h with LZ-1105 administration.

File Name: Supplementary Movie.4

Description: Ischemic reperfusion in hindlimb after clipping for 12 h with LZ-1105 administration.

File Name: Supplementary Movie.5

Description: Thrombolytic process in carotid thrombosis mice model with LZ-1105 administration.

File Name: Supplementary Movie.6.

Description: Open and recovery of blood brain barrier of mice with LZ-1105 administration.
