# Supplementary material for: Organic NIR-II molecule with long blood half-life for in vivo dynamic vascular imaging
Source: Nat Commun. 2020 Jun 18;11:3102. doi: 10.1038/s41467-020-16924-z (PMC7303218; doi:10.1038/s41467-020-16924-z)
Supplement: Supplementary file 10 — Reporting Summary [file 41467_2020_16924_MOESM10_ESM.pdf]

nature research

Corresponding author(s): Fan Zhang

Reporting Summary

Nature Research wishes to improve the reproducibility of the work that we publish. This form provides structure for consistency and transparency in reporting. For further information on Nature Research policies, see Authors & Referees and the Editorial Policy Checklist.

Statistical parameters

When statistical analyses are reported, confirm that the following items are present in the relevant location (e.g. figure legend, table legend, main text, or Methods section).

n/a	Confirmed	
☐	☒	The exact sample size (n) for each experimental group/condition, given as a discrete number and unit of measurement
☐	☒	An indication of whether measurements were taken from distinct samples or whether the same sample was measured repeatedly
☒	☐	The statistical test(s) used AND whether they are one- or two-sided<br>*Only common tests should be described solely by name; describe more complex techniques in the Methods section.*
☒	☐	A description of all covariates tested
☒	☐	A description of any assumptions or corrections, such as tests of normality and adjustment for multiple comparisons
☐	☒	A full description of the statistics including central tendency (e.g. means) or other basic estimates (e.g. regression coefficient) AND variation (e.g. standard deviation) or associated estimates of uncertainty (e.g. confidence intervals)
☒	☐	For null hypothesis testing, the test statistic (e.g. F, t, r) with confidence intervals, effect sizes, degrees of freedom and P value noted<br>*Give P values as exact values whenever suitable.*
☒	☐	For Bayesian analysis, information on the choice of priors and Markov chain Monte Carlo settings
☒	☐	For hierarchical and complex designs, identification of the appropriate level for tests and full reporting of outcomes
☒	☐	Estimates of effect sizes (e.g. Cohen's d, Pearson's r), indicating how they were calculated
☐	☒	Clearly defined error bars<br>*State explicitly what error bars represent (e.g. SD, SE, CI)*

Our web collection on statistics for biologists may be useful.

Software and code

Policy information about availability of computer code

Data collection	Images were acquired with Lightfield 6.11 software.
Data analysis	Statistical analyses were performed using OriginPro 8.0 and Microsoft Excel 2010 software. Images were processed with Matlab R2015b software. Chemical structures were created using ChemBioDraw Ultra 12.0. Density function theory (DFT) calculations was performed using Gaussian 03 revision C.02 software andB3LYP method and 6-31G* basis set. The kinetic study for LZ-1105 and ICG was fitted by Pharmacokinetic simulation software Drug and Statistics (DAS) 2.0.

For manuscripts utilizing custom algorithms or software that are central to the research but not yet described in published literature, software must be made available to editors/reviewers upon request. We strongly encourage code deposition in a community repository (e.g. GitHub). See the Nature Research guidelines for submitting code & software for further information.

Data

Policy information about availability of data

All manuscripts must include a data availability statement. This statement should provide the following information, where applicable:
- Accession codes, unique identifiers, or web links for publicly available datasets
- A list of figures that have associated raw data
- A description of any restrictions on data availability

The data that support the findings of this study are available from the corresponding authors upon reasonable request.

Field-specific reporting

Please select the best fit for your research. If you are not sure, read the appropriate sections before making your selection.

[x] Life sciences [ ] Behavioural & social sciences [ ] Ecological, evolutionary & environmental sciences

For a reference copy of the document with all sections, see nature.com/authors/policies/ReportingSummary-flat.pdf

Life sciences study design

All studies must disclose on these points even when the disclosure is negative.

Sample size	The sample size (n) of each experiment is provided in the corresponding figure captions in the paper and supplementary information. Sample sizes were chosen to support meaningful conclusions.
Data exclusions	No data were excluded from the analyses.
Replication	Animal experiments were performed on biological replicates following identical procedures to verify the reproducibility of the experimental findings.
Randomization	All samples/organisms were randomly allocated into experimental groups.
Blinding	Investigators were not blinded to group allocation during data collection and analysis.

Reporting for specific materials, systems and methods

Materials & experimental systems

n/a	Involved in the study	
x		Unique biological materials
x		Antibodies
	x	Eukaryotic cell lines
x		Palaeontology
	x	Animals and other organisms
x		Human research participants

Methods

n/a	Involved in the study	
x		ChIP-seq
x		Flow cytometry
x		MRI-based neuroimaging

Eukaryotic cell lines

Policy information about cell lines

Cell line source(s)	Human umbilical vein endothelial cells (HUVEC) were purchased from Stem Cell Bank, Chinese Academy of Sciences.
Authentication	All cell lines were authenticated by Short Tandem Repeat test.
Mycoplasma contamination	All cell lines tested negative for mycoplasma contamination.
Commonly misidentified lines (See ICLAC register)	No commonly misidentified cell lines were used in the study.

Animals and other organisms

Policy information about studies involving animals; ARRIVE guidelines recommended for reporting animal research

Laboratory animals	Female Balb/c nude mice (5 weeks old, ~15 g) and female ICR mice (5 weeks old, ~20 g) were obtained from Shanghai SLAC Laboratory Animal Centre (China). Mice were housing in independent ventilation cage (IVC) system under specific-pathogen free (SPF) condition. Housing temperature was 22-25 ℃, humidity was 35-45 %. The dark/light cycle was 12 h light/12 h dark.
Wild animals	The study did not involve wild animals.
Field-collected samples	The study did not involve sampled collected from the field.
